# Genome-wide Analysis of Phospho*enol*pyruvate Carboxylase Gene Family and Their Response to Abiotic Stresses in Soybean

**DOI:** 10.1038/srep38448

**Published:** 2016-12-07

**Authors:** Ning Wang, Xiujuan Zhong, Yahui Cong, Tingting Wang, Songnan Yang, Yan Li, Junyi Gai

**Affiliations:** 1 National Key Laboratory of Crop Genetics and Germplasm Enhancement/National Center for Soybean Improvement/Key Laboratory for Biology and Genetic Improvement of Soybean (General, Ministry of Agriculture)/Jiangsu Collaborative Innovation Center for Modern Crop Production, Nanjing Agricultural University, Nanjing, Jiangsu, 210095, China

## Abstract

Phospho*enol*pyruvate carboxylase (PEPC) plays an important role in assimilating atmospheric CO_2_ during C_4_ and crassulacean acid metabolism photosynthesis, and also participates in various non-photosynthetic processes, including fruit ripening, stomatal opening, supporting carbon–nitrogen interactions, seed formation and germination, and regulation of plant tolerance to stresses. However, a comprehensive analysis of PEPC family in *Glycine max* has not been reported. Here, a total of ten *PEPC* genes were identified in soybean and denominated as *GmPEPC1*-*GmPEPC10*. Based on the phylogenetic analysis of the PEPC proteins from 13 higher plant species including soybean, PEPC family could be classified into two subfamilies, which was further supported by analyses of their conserved motifs and gene structures. Nineteen *cis*-regulatory elements related to phytohormones, abiotic and biotic stresses were identified in the promoter regions of *GmPEPC* genes, indicating their roles in soybean development and stress responses. *GmPEPC* genes were expressed in various soybean tissues and most of them responded to the exogenously applied phytohormones. *GmPEPC6*, *GmPEPC8* and *GmPEPC9* were significantly induced by aluminum toxicity, cold, osmotic and salt stresses. In addition, the enzyme activities of soybean PEPCs were also up-regulated by these treatments, suggesting their potential roles in soybean response to abiotic stresses.

The enzyme phospho*enol*pyruvate (PEP) carboxylase (PEPC; EC 4.1.1.31) catalyzes the irreversible β-carboxylation of PEP in the presence of bicarbonate (HCO_3_^−^) and Mg^2+^ or Mn^2+^ to yield oxaloacetate (OAA) and inorganic phosphate (Pi)^1^. It is widely distributed in archaea, bacteria, cyanobacteria, green algae, protozoa, and vascular plants, but is absent from animals and fungi^1^,^2^. In plants, the reaction catalyzed by PEPC is the primary fixation step of photosynthetic CO_2_ assimilation in C_4_ photosynthesis and crassulacean acid metabolism (CAM). In most nonphotosynthetic tissues and in the leaves of C_3_ plants, PEPC plays the anaplerotic role of replenishing tricarboxylic acid (TCA) cycle with intermediates that are consumed for a variety of biosynthetic pathways and nitrogen assimilation^1^,^2^. Therefore, besides its fundamental role in the initial fixation of atmospheric CO_2_ during C_4_ and CAM photosynthesis, PEPC has a wide range of non-photosynthetic roles including supporting carbon–nitrogen interactions^2^, seed formation and germination^2^,^3^, fruit ripening^2^, guard cell metabolism during stomatal opening^4^, and provision of malate as a respiratory substrate for symbiotic N_2_-fixing bacteroids in legume root nodules^5^. Recently, accumulating evidence has confirmed that a large number of *PEPC* genes are induced by abiotic and biotic stresses and play important roles in regulation of plant tolerance to stress^2^,^6^,^7^. For example, an up-regulated PEPC expression in response to salinity or drought stress has been well documented in C_3_, C_4_ and CAM plants^6^,^7^,^8^,^9^,^10^. Meanwhile, overexpression of *PEPC* gene in transgenic plants enhanced their tolerance to drought or salt stress^11^, whereas knockdown of *PEPC* leading to increased sensitivity to osmotic stress^12^.

Plants have two types of PEPCs that belong to different evolutionary lineages, namely, plant-type PEPC (PTPC) isozyme and bacterial-type PEPC (BTPC) isozyme types^1^,^3^,^13^. Previous studies showed *PTPC* genes encode highly closely related 100–110 kDa polypeptides that contain a conserved N-terminal seryl-phosphorylation site and typically exist as homotetrameric Class-1 PEPCs^1^,^2^. It is suggested that all PTPCs evolved from a common ancestral gene and display a high degree of conservation at the genetic level^14^. *BTPC* genes encode larger 116–118 kDa polypeptides that exhibit low (40%) sequence identity with PTPCs, and contain a prokaryotic-like (R/K) NTG C-terminal tetrapeptide^3^. Although key residues and domains involved in the catalytic reaction and binding of substrates and inhibitors are conserved in both PTPCs and BTPCs, BTPCs lack the distinctive N-terminal serine phosphorylation motif of PTPCs, and appear to only exist as catalytic and regulatory subunits of extraordinary heteromeric complexes (Class-2 PEPCs)^3^.

Since the release of whole-genome sequences of many plant species, numerous PEPC proteins have been successfully identified and investigated in plants including *Arabidopsis thaliana*^13^, *Hordeum vulgare*^15^, *Lotus japonicus*^5^, *Solanum lycopersicum*^16^, *Solanum tuberosum*^17^, and *Triticum aestivum*^18^. For instance, four *PEPC* genes (*PEPC1- PEPC4*) were characterized in *A. thaliana*^13^. Three dicot C_4_ *PEPC* (*ppc-A*, *ppc-B*, *ppc-C*) genes were analyzed in *Flaveria* (Asteraceae), with the *ppc-A* gene being identified as the gene recruited for use in the C_4_ photosyntehic pathway^19^. In grasses, there are four *PEPC* genes that have been predominantly characterized, and *ppc-C*_*4*_ and *ppc-B2* are often recruited for use in C_4_ photosynthesis^20^. In sedges, there are five *PEPC* genes that have been predominantly characterized, with the *ppc-1* gene being recruited for use in C_4_ photosynthesis^21^.

Soybean (*Glycine max*) is one of the most important crops for vegetable protein and oil worldwide, and its capacity to fix atmospheric nitrogen through symbioses with soil-borne microorganisms would help sustainable agriculture development. Previously, five *PEPC* genes including *GmPEPC1*^22^ (also termed *GmPEPC15*^23^), *GmPEPC4*^23^, *GmPEPC7*^23^, *GmPEPC16*^24^, and *GmPEPC17*^25^ have been identified in soybean. The release of the complete soybean genome sequences makes it possible to identify genome-wide *PEPC* genes in soybean^26^. To get a more comprehensive understanding of the *PEPC* gene family in soybean, a genome-wide identification and characterization of soybean *PEPC*s was performed. Their phylogenetic relationships and protein motifs were analyzed together with the PEPCs from 12 other plant species. In addition, their expression patterns in deferent organs and in response to various abiotic stresses and hormones were also investigated, including aluminum (Al), cold, salt, and osmotic stresses as well as exogenously applied abscisic acid (ABA), aminocyclopropane carboxylic acid (ACC), gibberellic acid (GA), and jasmonic acid (JA). To our knowledge, this is the first study on genome-wide analysis of the soybean *PEPC* gene family, which would provide valuable information for further functional investigations of soybean PEPC.

## Results

### Identification and phylogenetic analysis of soybean *PEPC* genes

Blast searches of the soybean genome (*G. max* var. Williams 82) were performed by using all of the four Arabidopsis *PEPC* sequences as baits, and a total of 10 putative *PEPC* genes were identified (Table 1). Their deduced proteins were subjected to domain analysis using Pfam and SMART databases. These 10 non-redundant genes were confirmed as *PEPC* and denominated as *GmPEPC1*-*GmPEPC10*, according to the soybean nomenclature based on physical locations. The detailed information of *GmPEPC* gene family members, including gene name, gene model name, protein ID, chromosomal position, length of open reading frame (ORF), as well as the size (number of amino acids), molecular weight (MW), isoelectric point (pI), and proposed subcellular localization of the deduced protein, is shown in Table 1. These 10 *GmPEPC* genes locate on six chromosomes in soybean, with three *GmPEPC* genes on chromosome 12, two *GmPEPC* genes on chromosome 6 and chromosomes 13, respectively, and one *GmPEPC* on Chromosome 2 and 12 each (Table 1).

**Figure 1:**
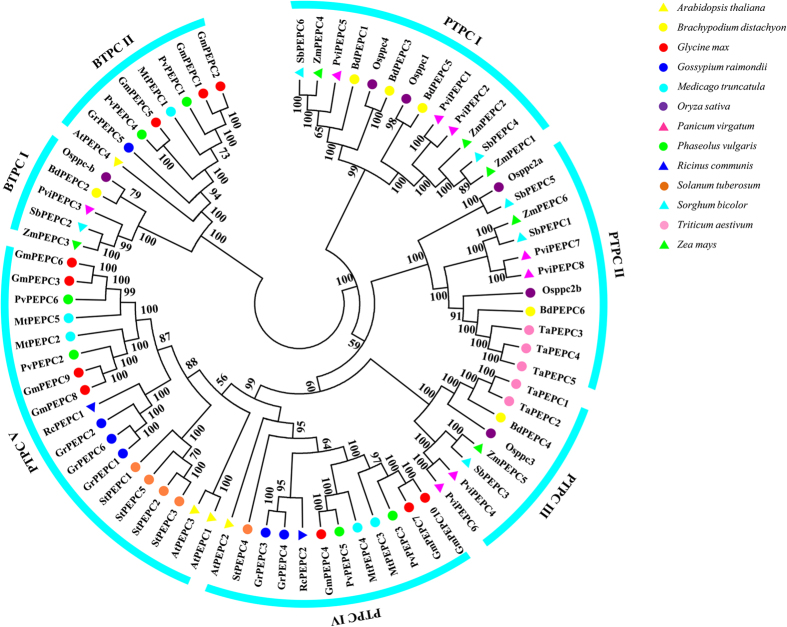
Phylogenetic analysis of PEPC proteins in soybean and other plant species. The full-length amino acid sequences of 75 PEPC proteins from 13 different plant species were used to construct the neighbor-joining tree using ClustalX 2.0 and MEGA 6.0 with 1000 bootstrap replicates. Branches with less than 50% bootstrap support were collapsed. The PEPCs were classified into two subfamilies (PTPC and BTPC). Subfamily PTPC is further separated into five clades from PTPC I to PTPC V, whereas subfamily BTPC is separated into two clades including BTPC I and BTPC II. Different plant species were distinguished by different colors.

**Figure 2:**
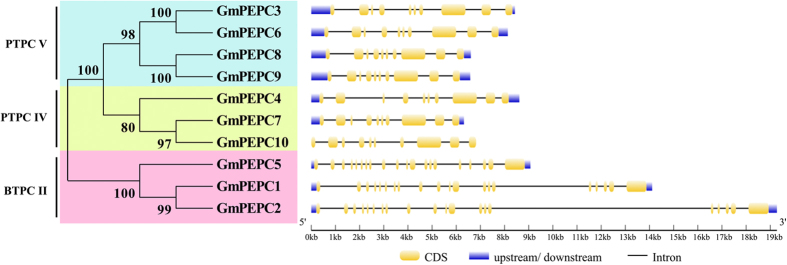
The phylogenetic relationship and exon-intron structures of PEPC family in soybean. Exon-intron structure was analyzed by online tool Gene Structure Display Server (GSDS). Lengths of exons and introns of *GmPEPC* genes were exhibited proportionally as indicated by the scale on the bottom. The classification of soybean PEPCs was indicated by the phylogenetic relationship on the left.

To investigate the evolutionary relationship between soybean PEPC proteins and known PEPCs from other species, a neighbor-joining (NJ) phylogenetic tree was constructed using 75 PEPC proteins from 13 different plant species (Fig. 1). The dendrogram showed that these PEPCs could be classified into two subfamilies (PTPC and BTPC), based on their sequence similarity. PTPC subfamily contains 61 members and is further separated into five groups (PTPC I to PTPC V) (Fig. 1). BTPC subfamily is further separated into two groups (BTPC I and BTPC II) (Fig. 1). The 10 GmPEPCs were assigned to three groups with high bootstrap values: *GmPEPC4*, *GmPEPC7*, and *GmPEPC10* in PTPC IV group; *GmPEPC3*, *GmPEPC6*, *GmPEPC8*, and *GmPEPC9* in PTPC V group; and *GmPEPC1*, *GmPEPC2*, and *GmPEPC5* in BTPC II group (Figs 1 and 2). Similar group distribution patterns of PEPCs were also found in *Gossypium raimondii*, *Medicago truncatula* and *Phaseolus vulgaris* (Table 2). The total number of PEPCs in soybean is the largest compared with the other 12 plant species (Table 2). Phylogenetic analysis also identified some closely related orthologous PEPCs between *G. max* and *P. vulgaris* (Fig. 1; Supplementary Figure S1): GmPEPC7, GmPEPC10 and PvPEPC3, GmPEPC4 and PvPEPC5, in PTPC IV group; GmPEPC3, GmPEPC6 and PvPEPC6, GmPEPC8, GmPEPC9 and PvPEPC2, in PTPC V group; GmPEPC1, GmPEPC2 and PvPEPC1, GmPEPC5 and PvPEPC4, in BTPC II group, suggesting that an ancestral set of PEPCs existed prior to the divergence of *G. max* and *P. vulgaris*.

### Gene structure and conserved motif of PEPC family

To obtain further insights into the possible structural evolution of *PEPC* genes, their exon-intron organizations were compared. As shown in Fig. 2, *GmPEPC* genes of different subfamilies exhibit different exon-intron structures. *GmPEPC* genes in PTPC V and PTPC IV contain 10 exons and 9 introns, while *GmPEPC* genes in BTPC II contain 20 exons and 19 introns. During the evolution of multigene families, diversification of gene structure is a common phenomenon, which may facilitate evolutionary cooption of genes for new functions to adapt to environmental changes. Different exon-intron structures between subfamilies were also observed for *PEPC* genes in both C_3_ (such as *G. max* and *Oryza sativa*) and C_4_ (such as *Zea mays* and *Sorghum bicolor*) plant species (Supplementary Figure S1), indicating conserved features of the gene structure of *PEPC* family. Meanwhile, the numbers of exons and introns are also conserved within each subfamily (Supplementary Figure S1). For example, most genes from PTPC subfamily usually contain 10 exons and 9 introns; most genes from BTPC subfamily contain 20 exons and 19 introns (Supplementary Figure S1). However, the sizes of plant *PEPC* genes vary dramatically, while the lengths of exons are similar within each subfamily, which suggests that the sizes of the genes depend largely on the sizes of the introns.

**Figure 3:**
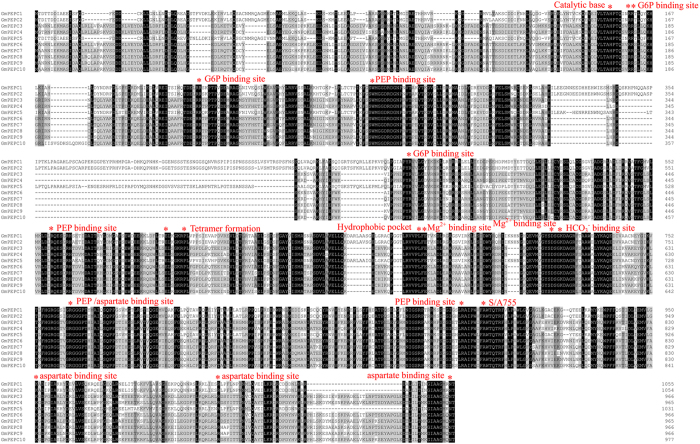
Multiple alignment of Soybean PEPC amino acid sequences. Black, gray, and light shading indicate 100%, 75%, and 50% similarities, respectively. Spots represent gaps. Amino acid residues of experimentally proven function are indicated by *above the alignment.

**Figure 4:**
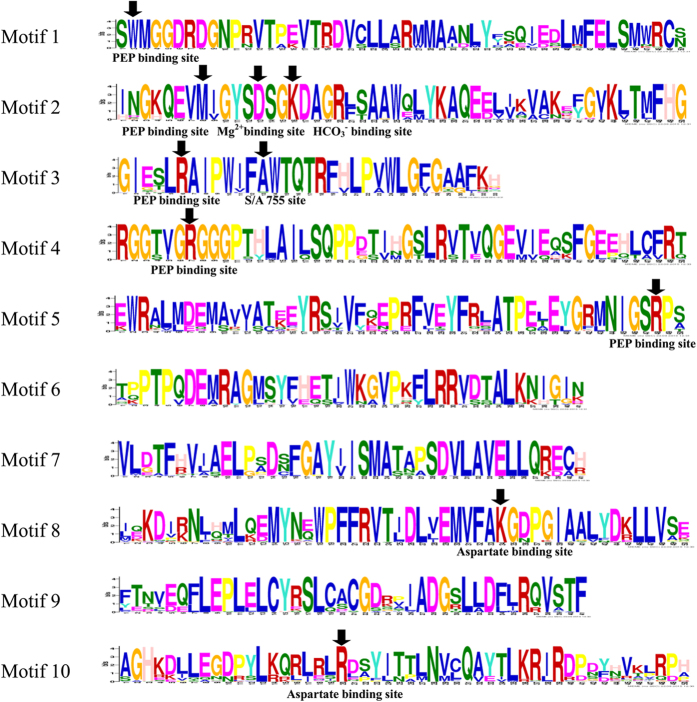
Conservation and diversity of the motifs in PEPC proteins. The schematic representation of ten motifs in PEPC family is elucidated by MEME. Amino acid residues of experimentally proven function are indicated by black arrows.

The GmPEPC protein sequences show highly conserved features of plant PEPC proteins (Fig. 3). The multiple alignments of the 10 soybean PEPC amino acid sequences showed 70.23% identical positions. The average identity scores of soybean PTPC proteins and soybean BTPC proteins were 92.30% and 92.44%, respectively. We predicted 10 most conserved motifs of plant PEPCs from all tested plant species using MEME software (Table 3; Fig. 4; Supplementary Figure S2). Results showed that PTPC proteins contain 10 motifs, and BTPC proteins contain 9 motifs (lacking motif 8, Supplementary Figure S2), which supported the phylogenetic relationship and classification of plant PEPCs. Notably, seven (1–5, 8 and 10) out of 10 motifs were annotated as key domains involved in the catalytic reaction and in the binding of substrates and inhibitors, which are conserved in both PEPC types (PTPC and BTPC). Additionally, we also analyzed PEPC motifs taking locations in the simulated structure into consideration. As shown in Fig. 4, five motifs contain PEP binding sites, including motif 1 to motif 5. These five PEPC motifs in all analyzed plant species showed the following patterns: motif 1 (SWMGGDRDGNP[RN]VT) has a highly conserved residue PEP binding site W; motif 2 (GKQEVM[IV]GYSDSGKDAGR[LF][ST]AAW) contains a PEP binding site M, also with a D774 that constitutes the binding site for Mg^2+^ and with a K777 that constitutes the binding site for HCO_3_^−^; Motif 3 (GI[EG][ST]LRAIPW[IY]F[AS]WTQ) has a PEP binding site R, also with a S/A that constitutes the binding site for S/A 755 site; Motif 4 (G[TS]YG**R**GGGP[TC][HY]L) and Motif 5 ([RI][ML]NIGS**R**P[SA]) only has a PEP binding site R. Motif 8 (EMVFA**K**G[DN]PG) and Motif 10 ([LI][REQ][LSIN]**R**[DLEN][SAP][YF]) only has an aspartate binding site K or R, respectively. Whereas another three unknown functional motifs of 6, 7 and 9 were found in all 75 plant PEPCs (Table 3; Fig. 4; Supplementary Figure S2).

### *cis*-elements in the promoter regions of *GmPEPC* genes

**Figure 5:**
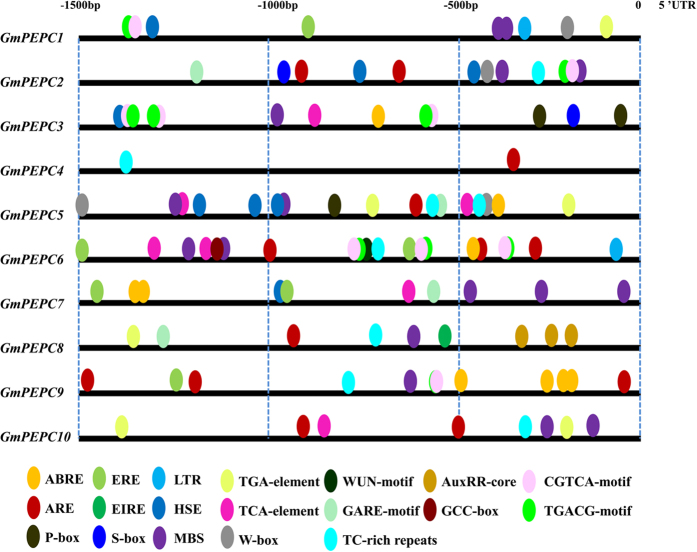
Predicted *cis*-elements in the promoter regions of *GmPEPC* genes. The 1500 bp promoter regions of 10 *GmPEPC* genes were analyzed to predict the *cis*-elements, which were presented as colored ellipses: ABA responsive element (ABRE), anaerobic responsive element (ARE), auxin responsive element (TGA-element, AuxRR-core), light-responsive element (G-box), gibberellin responsive element (GARE-motif), ethylene responsive element (ERE, GCC-box), heat stress responsive element (HSE), low-temperature-responsive element (LTR), MYB binding site (MBS), pathogen-related *cis*-element (S-box), defense and stress-responsive element (TC-rich repeats), salicylic acid responsive element (TCA-element), jasmonic acid responsive element (TGACG-motif, CGTCA-motif), wound-responsive element (WUN-motif), endosperm development (P-box), and WRKY binding site (W-box). The numbers on the top indicate the relative positions to the start codon.

It has been reported that a large number of *PEPC* genes are induced by abiotic and biotic stresses and play important roles in the regulation of plant tolerance to stresses^2^,^6^,^7^. To investigate the possible roles of PEPCs identified in the soybean genome, the 1500 bp sequences upstream of the start codon of *GmPEPC* genes were used to analyze *cis*-elements in their promoter regions by PlantCARE (Fig. 5; Supplementary Table S4). Nineteen putative *cis*-elements responsive to biotic stresses [including EIRE (elicitor-responsive element^27^), S-box (pathogen-related *cis*-element^28^), W-box^29^, TC-rich repeats (defense and stress-responsive element^30^), and WUN-motif (wound-responsive element^31^)], abiotic stresses [ARE (anaerobic-responsive element), HSE (heat stress-responsive element^32^), LTR (low-temperature responsive element^33^), MBS (MYB binding site^34^)], and phytohormones such as ABA [abscisic acid, ABRE (ABA responsive element^35^)], auxin (TGA-element^36^; AuxRR-core^37^), ET [ethylene, ERE (ethylene-responsive element^38^ and GCC-box^39^)], GA (gibberellin, GARE^40^), JA (jasmonic acid, CGTCA-motif^41^ and TGACG-motif^42^), SA (salicylic acid, TCA-element^43^) and endosperm development (P-box^44^) were present in the promoters of *GmPEPC* genes (Fig. 5, Supplementary Table S4). There are other *cis*-elements (Supplementary Table S4) such as light responsive elements (Box I^45^, GATA-motif^46^ and GT1-motif^47^) were also present in the promoter regions of *GmPEPC* genes. These results provide further support for the diverse roles of *GmPEPC* genes in soybean developmental processes, as well as response to biotic and abiotic stresses.

### Expression patterns of *GmPEPC* genes in different soybean tissues

**Figure 6:**
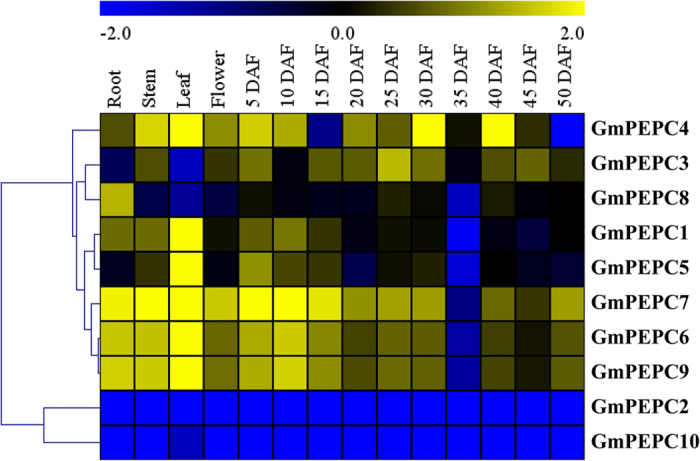
Expression patterns of *GmPEPC* genes in different soybean tissues. The expression patterns of 10 *GmPEPC* genes in 14 soybean tissues were investigated by qRT-PCR, using soybean *GmRP15* gene as the internal control. Root, stem, leaf, flower, and seeds from different development stages were subjected to analysis. DAF: day after flowering. The experiments were repeated three times, log 2 based value was used to create the heat map with clustering of genes. The expression levels are shown from the lowest (blue) to highest (yellow) in heat colors as indicated by the scale on the top.

The expression patterns of *GmPEPC* genes in soybean different tissues were analyzed by qRT-PCR. The results showed that these 10 *GmPEPC* genes were expressed with different tissue-specific patterns (Fig. 6). For example, *GmPEPC4* was expressed higher in stem, leaf, flower, and younger seed but lower in the seed of 15 days after flowering (DAF) and 50 DAF. Both *GmPEPC3* and *GmPEPC8* were expressed at lower levels in soybean leaf, but *GmPEPC3* showed relatively higher expression levels during seed development, while *GmPEPC8* expressed higher in root. Both *GmPEPC1* and *GmPEPC5* showed higher expression in soybean leaf but low in seed at 35 DAF stage. *GmPEPC6*, *GmPEPC7* and *GmPEPC9* showed high expression levels in all tested soybean tissues except seed of 35 DAF. The expression levels of *GmPEPC2* and *GmPEPC10* were low in all tested tissues (Fig. 6).

### Expression profiles of *GmPEPC* genes in response to abiotic stresses and phytohormones

**Figure 7:**
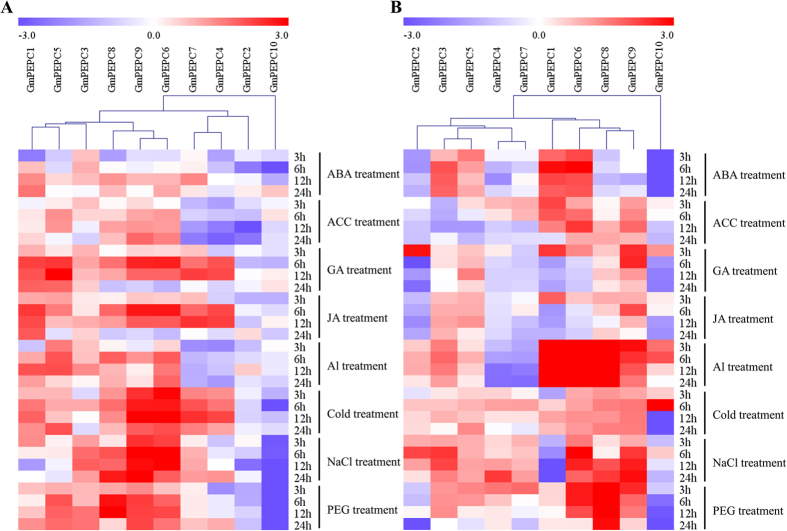
Expression profiles of *PEPC* genes in leaves (**A**) and roots (**B**) of soybean in response to ABA, ACC, GA, JA, Al, cold, salt (NaCl) and osmotic (PEG) treatments. The relative expression levels of the 10 *PEPC* genes were quantified by qRT-PCR, using soybean *GmRP15* gene as the internal control. Leaves and roots of 14-d soybean seedlings are used to investigate the changes in PEPC expression under different treatments, including 100 μM ABA (Abscisic acid), 100 μM ACC (aminocyclopropane carboxylatesythase), 100 μM GA (Gibberellin), 100 μM JA (Jasmonic acid), 25 μM AlCl_3_ (pH 4.3), 4 °C cold, 200 mM NaCl, and 20% PEG6000. The experiments were repeated three times, log2 based value (fold change) was used to create the heat map with clustering of genes. All data were normalized to the expression level of control (0 h). The scale represents the relative expression levels from low (blue) to high (red).

The presences of stress-responsive *cis*-elements in the promoter regions of the *GmPEPC* genes suggest their involvement in soybean response to different stresses. To further investigate the possible functions of soybean *PEPC* genes, the transcriptional expression of 10 *GmPEPC* genes in soybean plants were analyzed by qRT-PCR analysis after exogenous application of ABA, ACC, GA, and JA as well as under Al toxicity, cold stress, osmotic stress, and salt stress (Fig. 7; Supplementary Figures S3 and S4). Almost all *GmPEPC* genes responded to the exogenously applied hormones and stress treatments, with the majority being upregulated most of the time, except *GmPEPC2*, *GmPEPC4* and *GmPEPC10*, which were non-responsive or downregulated at most of the tested time points under these treatments (Fig. 7; Supplementary Figures S3 and S4). In soybean leaves (Fig. 7A), under ABA treatment, the relative expression levels of *GmPEPC1*, *GmPEPC3*, *GmPEPC6*, and *GmPEPC8* were induced at least two time points, while *GmPEPC4*, *GmPEPC5* and *GmPEPC10* showed decreased transcripts at least two time points. After ACC treatment, *GmPEPC1*, *GmPEPC5*, *GmPEPC6*, *GmPEPC8*, and *GmPEPC9* were substantially upregulated. The increase in their levels started as early as 3 h after ACC stress and continued till 24 h of stress. On the other hand, several *GmPEPC* genes (*GmPEPC2*, *GmPEPC4*, and *GmPEPC7*) were significantly downregulated during ACC stress. With GA treatment, except *GmPEPC2* and *GmPEPC10* were significantly downregulated at 6 h, all of the rest *GmPEPC* genes were induced at least two time points. The expression profile of soybean *PEPC* genes under JA treatment is similiar with GA stress, except *GmPEPC2* and *GmPEPC10*, which were down-regulated, and all of the rest genes were induced at least two time points. Under Al stress, the expression of *GmPEPC1*, *GmPEPC3*, *GmPEPC5*, *GmPEPC6* and *GmPEPC8* was induced at most of the time points, while the expression of *GmPEPC9* was only induced at 6 h. The relative expression of *GmPEPC4* and *GmPEPC7* were inhibited under Al stress. Most *GmPEPC* genes, except for *GmPEPC2*, *GmPEPC3* and *GmPEPC10*, were upregulated during cold stress treatment. Under salt stress, the transcript abundance of *GmPEPC3*, *GmPEPC6*, *GmPEPC8*, and *GmPEPC9* increased in at least three time points, while *GmPEPC10* decreased across all time points (Fig. 7A; Supplementary Figure S3). Water-deficit conditions were imposed by transferring the soybean seedlings to 20% PEG solution and the expression levels of *GmPEPC* genes were examined during stress. *GmPEPC3*, *GmPEPC5*, *GmPEPC6*, *GmPEPC8*, and *GmPEPC9* showed induced expression in at least two time points whereas *GmPEPC2* and *GmPEPC10* showed downregulation across all time-points. The cluster analysis showed that *GmPEPC6*, *GmPEPC8*, and *GmPEPC9* were grouped together and showed more responsive to abiotic stresses.

We also analyzed the expression patterns of *GmPEPC* genes in soybean roots under phytohormone and abiotic treatments (Fig. 7B; Supplementary Figure S4). The transcript levels of four *GmPEPC* genes (*GmPEPC1*, *GmPEPC3*, *GmPEPC*5, and *GmPEPC*6) can be significantly induced in response to exogenous application of ABA, while all other genes especially *GmPEPC10* are downregulated by ABA. *GmPEPC1*, *GmPEPC6* and *GmPEPC9* were highly upregulated by ACC. However, the expression levels of *GmPEPC2* and *GmPEPC3* declined in at least three time points. With GA treatment, *GmPEPC5*, *GmPEPC8*, and *GmPEPC9* were induced while *GmPEPC7* was downregulated during the whole treatment. Under JA treatment, the transcript levels of four *GmPEPC* genes (*GmPEPC3*, *GmPEPC5*, *GmPEPC8*, and *GmPEPC9*) can be significant induced, while *GmPEPC2* showed significant downregulation across all time points. Under Al stress, except for *GmPEPC4* and *GmPEPC7*, the expression levels of all other eight *GmPEPC* genes were increased. Under cold stress, the mRNA levels of most *GmPEPC* genes (except *GmPEPC10*) exhibited significantly upregulated in most time points. Under salt stress, the transcript levels of eight *GmPEPC* genes were upregulated except *GmPEPC1* and *GmPEPC10*. When treated with 20% PEG, the expression of *GmPEPC6*, *GmPEPC8*, and *GmPEPC9* was induced significantly, while *GmPEPC2* and *GmPEPC10* showed down-regulation (Fig. 7B; Supplementary Figure S4). Three genes, *GmPEPC6*, *GmPEPC8*, and *GmPEPC9* showed significant induced expression in soybean roots under abiotic stresses and were cluster together, which is consistent with their expression patterns in soybean leaves (Fig. 7).

### PEPC activity in response to abiotic stresses and phytohormones

**Figure 8:**
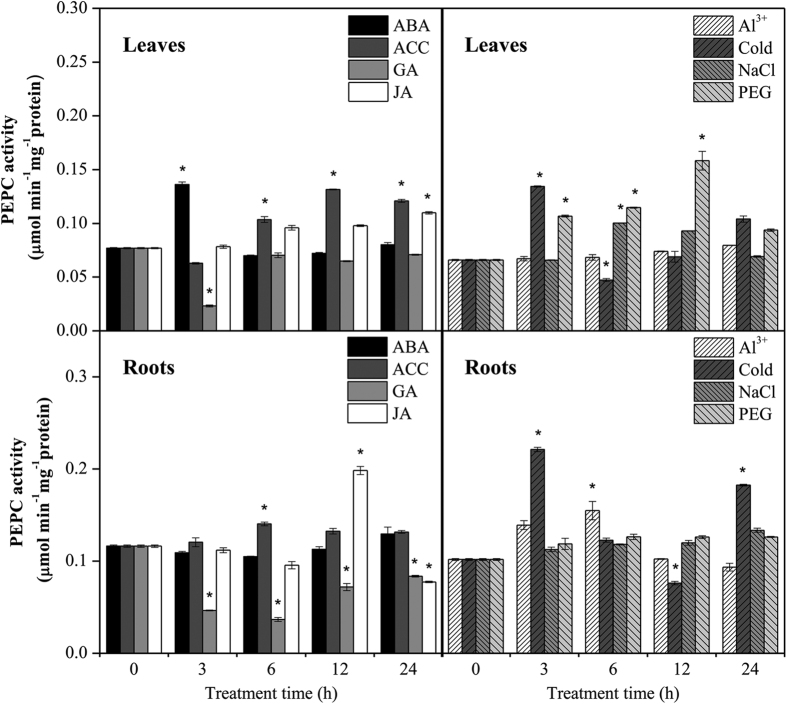
PEPC activities in soybean leaves and roots subjected to different abiotic and phytohormone treatments. Leaves and roots of 14-d soybean seedlings are used to investigate the changes in PEPC activities under different treatments, including 100 μM ABA (Abscisic acid), 100 μM ACC (aminocyclopropane carboxylatesythase), 100 μM GA (Gibberellin), 100 μM JA (Jasmonic acid), 25 μM AlCl_3_ (pH 4.3), 4 °C cold, 200 mM NaCl, and 20% PEG6000. Data shown are means ± SD of three independent experiments. Statistical significance of differences between control and treated groups was analyzed using Student’s t-test (*indicates *P* < 0.05).

To further investigate the potential functions of *GmPEPC* genes in soybean response to abiotic stresses, the enzyme activities of GmPEPCs in soybean leaves and roots under different phytohormones and abiotic treatments were assayed. As shown in Fig. 8, ABA and ACC treatments significantly increased the PEPC activity compared with control in soybean leaves. JA treatment resulted in a significant increased PEPC activity in both leaves and roots. Under abiotic stresses, the PEPC activity in leaves was significantly increased by cold, PEG, and salt stresses, especially PEG treatment. In soybean roots, the PEPC activity was significantly upregulated by cold and Al stresses.

## Discussion

PEPC plays an important role in assimilating atmospheric CO_2_ during C_4_ and CAM photosynthesis, and also participates in various non-photosynthetic processes. However, a comprehensive analysis of *GmPEPC* genes in soybean has not been reported. In the present study, a total of ten PEPCs were identified in the soybean genome from Phytozome database^48^. The numbers of *PEPC* genes in soybean were slightly more than that in other plant species, such as *A.thaliana* (4 *PEPC* genes), *Brachypodium distachyon* (6 *PEPC* genes), *G. raimondii* (6 *PEPC* genes), *M. truncatula* (5 *PEPC* genes), *O. sativa* (6 *PEPC* genes), *Panicum virgatum* (8 *PEPC* genes), *P. vulgaris* (6 *PEPC* genes), *Ricinus communis* (2 *PEPC* genes), *S. tuberosum* (5 *PEPC* genes), *S. bicolor* (6 *PEPC* genes), *T. aestivum* (5 *PEPC* genes), and *Z. mays* (6 *PEPC* genes) (Table 2). As the soybean genome is larger than the genomes of rice and Arabidopsis, it is possible that the soybean genome contains more *PEPC* genes than them. Interestingly, the genome size of soybean is smaller than maize and sorghum, but it also contains more *PEPC* genes. This might be due to the two ancient genome duplication events happened in soybean around 59 and 13 million years ago, which resulted in multiple copies of around 75% of its genes^26^.

Phylogenetic analyses of higher plant *PEPC* genes has been previously carried out in families of Cyperaceae, Poaceae, and Molluginaceae to determine which *PEPC* gene is recruited for use in C_4_ biochemistry^20^,^49^,^50^. Plants have two types of PEPCs including plant-type PEPC (PTPC) and bacterial-type PEPC (BTPC)^1^,^3^,^13^. In the core eudicots, there are two primary *PEPC* gene lineages that have been studied to date: *ppc-1*(*PTPC*) and *ppc-2* (*BTPC*)^50^. For example, in Arabidopsis, there are four *PEPC* genes, including three *PTPC* and one *BTPC*^13^. In rice, five rice *PEPC* genes (*Osppc1*, *2a*, *2b*, *3*, and *4*) encode the PTPCs and the other (*Osppc-b*) encodes a BTPC. In addition, all plant genomes sequenced to date, including that of ancestral green algae, contain at least one *BTPC* gene^2^. In this study, soybean *PEPC* genes were also distributed into two subfamilies (PTPC and BTPC), with seven genes (*GmPEPC3*, *GmPEPC4*, *GmPEPC6*, *GmPEPC7*, *GmPEPC8*, *GmPEPC9*, and *GmPEPC10*) in PTPC subfamily, and three genes (*GmPEPC1*, *GmPEPC2*, and *GmPEPC5*) in BTPC group. Moreover, the gene structures of *PTPC* and *BTPC* genes were different: *PTPC* genes have a highly conserved genomic structure composed of approximately ten exons, whereas *BTPC* genes have a very different and more complex structure with approximately twenty exons^1^. Similar results were also observed in this study, where *GmPEPCs* in PTPC subfamily contain 10 exons, and *GmPEPCs* in BTPC subfamily contain 20 exons (Fig. 2). We predicted 10 most conserved motifs in 75 PEPCs from 13 different species (Fig. 4; Supplementary Figure S3), and the ten PEPCs in soybean also possessed 10 conserved motifs (Table 3; Fig. 4). These data, together with previous studies, suggest similar origins and evolution patterns of the *PEPC* genes in different species.

*PEPC* genes have been reported in a wide range of tissues, including cell cultures and seedlings of *A. thaliana*^51^, seeds of *H. vulgare* and *R. communis*^15^, root nodules of *L. japonicus* and *G. max*^5^,^52^, cell cultures of *S. lycopersicum*^16^, seedlings of *S. tuberosum*^17^ and leaves of *T. aestivum*^18^. Besides, PEPC has a wide range of non-photosynthetic roles including seed formation and germination, fruit ripening, guard cell metabolism during stomatal opening^2^. Former study showed that the soybean bacterial-type gene *GmPEPC17* (termed *GmPEPC5* in this study) was expressed mainly in aboveground organs^25^. We also noticed that a higher expression level of *GmPEPC5* in stem and leaf, but not in the flower and root. Besides, the other two *GmBTPC* genes (*GmPEPC1* and *GmPEPC2*) have no or low expression in flowers (Fig. 6), which is consistent with the previous report that Arabidopsis *BTPC* transcripts were detected at low levels in siliques and flowers^13^. It is noteworthy that almost no detectable expression level of *GmPEPC2* was found in the examined tissues (Fig. 6). On the other side, three *GmPTPC* genes (*GmPEPC6*, *GmPEPC7* and *GmPEPC9*) expresses at relatively higher levels in most tissues and showed a similar expression pattern under normal condition (Fig. 6), whereas these genes expressed differently as reported before^25^. In addition, *GmPEPC8* is expressed at low levels in the tissues tested, except a relative higher level in root (Fig. 6), whereas the broad but low-level expression pattern of this gene has been reported^25^.

Extensive studies suggested *PEPC* genes play important roles in plant response to abiotic stresses. For example, studies have shown that *PEPC* overexpressing transgenic rice has a relatively higher photosynthetic rate under high light and temperature conditions^53^. In addition, abiotic stresses, such as salt and chilling injury, can induce *PEPC* gene expression in wheat, Arabidopsis and sorghum^8^,^10^,^54^. In this study, we showed that the promoter regions of most *GmPEPC* genes contained 19 stress-responsive *cis*-regulated elements such as ABA, cold, drought, heat, salt, and wound-related elements, indicating their potential roles in soybean response to phytohormone and stresses (Fig. 5; Supplementary Table S4). Previous reports also demonstrated the crucial roles of *PEPC* genes in various stress responses and in hormone signaling transduction^2^,^7^,^8^,^9^,^10^, which motivated us to perform expression profile analyses of *GmPEPC* genes under various abiotic stress and hormones treatments. The results showed that Al, cold, salt or PEG stress could alter the expression level of soybean *PEPC* genes and soybean PEPC activity (Figs 7 and 8). *GmPEPC6*, *GmPEPC8* and *GmPEPC9* were significantly induced by Al, cold, salt or osmotic stress, suggesting that these three *GmPEPC* genes are important for soybean response to abiotic stresses (Fig. 7). It has been reported that the exudation of PEPC-derived organic acids functions to chelate metals in the rhizosphere to alleviate heavy metal toxicity by preventing their uptake into the cell^2^. Overexpression of a C_4_ *PEPC* isoenzyme in rice led to increased oxalate exudation and Al tolerance^55^. In our study, eight out of ten *GmPEPC* genes in soybean roots were responsive to Al stress (Fig. 7B). On the other hand, although the up-regulation of *PEPC* genes has been well documented in plants^2^, we still found that a few *GmPEPC* genes (such as *GmPEPC2*, *GmPEPC4* and *GmPEPC10*) showed down-regulating or non-responsive tendency during the abiotic and phytohormone treatments. The down-regulation or non-responsiveness of *GmPEPC2*, *GmPEPC4* and *GmPEPC10* might be due to the low transcript abundance of *GmPEPC2* and *GmPEPC10* in all tested soybean tissues (Fig. 6) and few (only two) *cis*-elements responsive to hormones or stresses in the promoter region of *GmPEPC4* (Fig. 5), which indicates their less importance in soybean response to these hormone and stress treatments. The possible biological meaning of this phenomenon could be that different *GmPEPC* genes have different regulation mechanisms and diverged functions. In addition, the PEPC activity in soybean leaves increased after salt or PEG treatment, and PEPC activity in soybean roots was upregulated in response to Al or cold stress (Fig. 8). These results suggested that PEPC might be involved in soybean response to abiotic stresses.

In vascular plants, PEPCs are strictly regulated enzymes by a variety of mechanisms due to their irreversible nature of the enzymatic reactions. PEPC is activated by its positive effector, glucose 6-phosphate, and inhibited by its negative effectors, malate, aspartate, and glutamate, as well as phosphorylation catalyzed by a specific Ca^2+^-independent serine/threonine kinase known as PPCK (PEPC protein kinase)^2^, and dephosphorylation by a PP2A (protein phosphatase 2 A)^1^,^2^,^56^. The control of reversible phosphorylation catalyzed by PPCK is an important mechanism that regulates the activity of PEPC^57^. In this reaction, phosphorylation catalyzed by PPCK changes the sensitivity of PEPC to its allosteric effectors^2^. Previously, four *PPCK* genes (*GmPPCK1*, Glyma.03G251400; *GmPPCK2*, Glyma.20G222600; *GmPPCK3*, Glyma.10G166600; *GmPPCK4*, Glyma.10G186000) have been characterized in soybean^58^. In order to investigate the relationship between GmPEPC activity and *GmPPCK* expression, we examined the expression patterns of *GmPPCK* genes in soybean leaves and roots in response to abiotic stress and phytohormone applications (Supplementary Figure S5). The results showed that the expression patterns of four *GmPPCK* genes were not same. For example, under ABA treatment, only *GmPPCK1* was upregulated in soybean leaves, suggesting different *GmPPCK* genes have different regulation mechanisms in specific tissues and environments. And the changes in GmPEPC activities (Fig. 8) were not consistent with the changes in the transcript abundance of *GmPPCK* genes (Supplementary Figure S5) under these phytohormone and abiotic stress treatment, which revealed the complexity of the regulation mechanisms of PEPC activity. Indeed, degradation of both PEPC and PPCK by the polyubiquitin–proteasome pathway has been reported^59^,^60^. Recently, monoubiquitination of PEPC during sorghum seed development and germination were also documented^61^. Additionally, the dephosphorylation of PEPC is catalyzed by PP2A^1^,^2^,^56^. In summary, our results suggest a complex regulation mechanism of GmPEPC activity.

In conclusion, we conducted a genome-wide survey of the PEPC family in soybean. In silico analysis of the soybean genome database identified 10 *PEPC* genes, supported by conserved domain and multiple sequence alignments. Phylogenetic analyses of 75 *PEPC* genes from 13 speices indicated that these PEPCs could be divided into two subfamilies. This classification was further supported by gene structure and motif analyses, with each group sharing common features of exon-intron and protein motifs. The *GmPEPC* genes were expressed in soybean roots, stems, leaves, flowers and developing seeds. The presence of important *cis*-regulatory elements related to various stresses in the promoter regions of *GmPEPC* genes indicates their putative functions in soybean response to stresses. Their transcript abundance and enzyme activities in soybean leaves and roots were altered by Al, cold, salt or PEG stress, as well as the exogenous application of ABA, GA, JA, and SA, implying that soybean PEPCs may participate in soybean response to abiotic stresses. Taken together, this work would provide a foundation for future functional investigation of the PEPC family in soybean.

## Methods

### Plant material and stress treatments

The seeds of soybean [*G. max* (L.) Merr.] variety ‘Kefeng No. 1’ (Al-tolerant and drought-tolerant cultivar)^62^,^63^ were obtained from the National Center for Soybean Improvement (Nanjing Agricultural University, Nanjing), and were sterilized in 3% (v/v) sodium hypochlorite for 10 min and rinsed thoroughly with deionized water. Seeds were placed on wet paper towels for 4 d in a plant growth chamber (Dongnan Equipment, Ningbo, China) with a 16-h-light/8-h-dark cycle at 28 °C/25 °C and a light intensity of 150 μmol· m^−2^·s^−1^. The chamber had a relative humidity of 70%. The seedlings were then transferred to plastic boxes (1 L) filled with half-strength Hoagland nutrient solution for hydroponic culture^64^ and incubated in the same growth chamber. The nutrient solution was changed every 3 days. For tissue-specific expression analysis, young leaves, stems, and roots were collected from 4-week-old seedlings at V2 stage; blooming flowers were sampled from plants at R2 stage, whereas developing seeds were collected since the beginning of R3 to R7 stages, at five days intervals^65^. For different abiotic stress and hormone treatment, 14-day-old seedlings were subjected to 25 μM AlCl_3_ (pH 4.3), 20% (w/v) polyethylene glycol (PEG) 6000, 200 mM NaCl, 4 °C cold treatment, 100 μM abscisic acid (ABA), 100 μM 1-aminocyclopropane-1-carboxylic acid (ACC), 100 μM jasmonic acid (JA), and 100 μM gibberellins (GA), respectively. Meanwhile, control plants were treated with half-strength Hoagland nutrient solution or 0.5 mM CaCl_2_ (pH = 4.3) solution containing either 0 μ M AlCl_3_ (control). Leaves and roots from all treatments were harvested separately at 0, 3, 6, 12, 24 and 48 h after treatment, then immediately frozen in liquid nitrogen and stored at −80 °C until use. Each sample was the mixture of three seedlings and each treatment was repeated three times.

### Identification and sequence analysis of *PEPC* genes

We searched the *PEPC* genes from thirteen plant genomes and their sequences and corresponding annotations were downloaded from Phytozome database (http://phytozome.jgi.doe.gov/pz/portal.html). We used the following steps to identify the *PEPC* genes. First, the amino acid consensus sequences of four Arabidopsis PEPC were used as queries to conduct BLASTP searches in the soybean Proteome (http://phytozome.jgi.doe.gov/pz/portal.html). The protein domain and motif analysis was performed using PFAM (http://pfam.sanger.ac.uk/), SMART (http://smart.emblheidelberg.de/) and MEME (http://meme.nbcr.net/meme/).

Multiple alignments of the sequences were performed using ClustalW with the default options^66^ in MEGA Version 6.0^67^. Phylogenetic trees were constructed based on the neighbor-joining (NJ) method with a Kimura 2-parameter model using MEGA v6.0. The stability of the internal nodes was assessed with a bootstrap analysis of 1,000 replicates.

### Gene structure analysis

The gene structures were predicted using the Gene Structure Display Server (http://gsds.cbi.pku.edu.cn/). Multiple expectation maximization for motif elicitation (MEME^68^) was employed to identify and analyze the conserved motifs of PEPC sequences in this study. Only the motifs with *P* values < 10^−6^ and no overlap with each other were reported.

### Analysis of *cis*-acting elements in *GmPEPC* promoter regions

The 1500 bp upstream sequences of the start codon were used to analyze the *cis*-elements in *GmPEPC* promoter regions using PlantCARE (http://bioinformatics.psb.ugent.be/webtools/plantcare/html/) and PLACE database^69^.

### Quantitative real-time PCR

Total RNA samples were extracted using RNeasy Plant Mini Kits with on-column DNase (RNase free DNase set) treatment (Qiagen, Dusseldorf, Germany). The RNA concentration and integrity were checked by spectrophotometry and gel electrophoresis. A total of 0.5 μg RNA per sample was reverse transcribed into cDNA with the PrimeScript™ II reverse transcription kit (TaKaRa, Dalian, China). The cDNAs were diluted 1:10 with nuclease-free water prior to the qRT-PCR analyses. Soybean *GmRP15* gene was used as the internal standard, and all gene-specific primers were designed using Primer-BLAST (http:// www.ncbi.nlm.nih.gov/tools/primer-blast/) and are listed in Supplementary Table S1. Quantitative real-time PCR (qRT-PCR) analysis was carried out with the IQ5 light cycler (Bio-Rad, Hercules, USA). Each PCR mixture contained 10 μL SYBR Premix Ex Taq II (TaKaRa), 0.5 μM gene-specific primers and 50–100 ng cDNA in a final volume of 20 μL. For tissue expression pattern analysis, the gene expression levels in different tissues were calculated using the 2^−ΔCt^ method^70^. The relative gene expression levels in response to various abiotic and phytohormone treatments were calculated using the 2^−ΔΔCt^ method^71^, using the control plants (0 h) as the reference. The expression heatmaps with clustering of genes were constructed by MeV 4.9 software with Euclidean distance metric using the average linkage method^72^.

### PEPC activity measurement

Approximately 0.5 g soybean leaves or roots were homogenized with 1.5 mL of extraction buffer containing 100 mM Tris–HCl (pH 7.8), 1 mM EDTA, 1 mM dithiothreitol (DTT), 5 mM MgCl_2_, 1% (w/v) polyvinylpyrrolidone (PVP), and 1% (v/v) protease inhibitor cocktail (Sigma, USA). Then the extract was centrifuged at 12000× rpm at 4 °C for 15 min. PEPC activity was measured at 30 °C by an enzyme-coupled spectrophotometric method^73^ with slight modifications. The standard assay mixture (a total volume of 1.0 ml) contained 100 mM HEPES-NaOH (pH 8.0), 0.2 mM NADH, 1 mM NaHCO_3_, 6 mM PEP, 10 mM MgCl_2_, and 12 units of malate dehydrogenase (MDH) from porcine heart mitochondria (Roche Diagnostics, Germany). PEPC activity was recorded by monitoring NADH oxidation at 340 nm in a spectrophotometer (Beckman DU200, Germany) at 25 °C. Assays were initiated by the addition of protein extracts. Protein amounts were determined by the method of Bradford^74^.

### Statistical analysis

Statistical analyses were performed with the SPSS version 17.0 software (SPSS, Chicago, IL, USA) for Windows. Data are presented as mean values ± SD of three independent experiments. Differences between treatments were analyzed using Student’s *t* test.

## Additional Information

**How to cite this article**: Wang, N. *et al*. Genome-wide Analysis of Phospho*enol*pyruvate Carboxylase Gene Family and Their Response to Abiotic Stresses in Soybean. *Sci. Rep.* **6**, 38448; doi: 10.1038/srep38448 (2016).

**Publisher’s note:** Springer Nature remains neutral with regard to jurisdictional claims in published maps and institutional affiliations.

## Supplementary Material

Supplementary Information

## Figures and Tables

**Table 1:** Characteristics and nomenclature of soybean *PEPC* genes and their encoded proteins.

Gene Name	Previously named	Gene Model Name (Wm82.a2.v1)	GenBank Protein ID	Chr	ORF (bp)	Protein			
						Size (amino acids)	Molecular Weight (Da)	isoelectric point (pI)	Subcellular localization
*GmPEPC1*		Glyma.01G091000	XP_006573282	1	3171	1056	118167.68	6.38	Cyto/Nucl
*GmPEPC2*		Glyma.02G130700	XP_014622374	2	3168	1055	118230.60	6.28	Cyto
*GmPEPC3*		Glyma.06G229900	XP_014632171	6	2904	967	110754.93	5.72	Cyto
*GmPEPC4*		Glyma.06G277500	XP_003527347	6	2901	966	110379.84	5.52	Cyto
GmPEPC5	*GmPEPC17*^25^	Glyma.10G205500	NP_001237378	10	3099	1032	115946.45	6.20	Cyto
GmPEPC6	*GmPEPC16*^24^	Glyma.12G161300	NP_001237602	12	2904	967	110790.15	5.66	Cyto
GmPEPC7	*GmPEPC4*^23^	Glyma.12G210600	NP_001237394	12	2901	966	110644.39	6.00	Cyto
GmPEPC8	*GmPEPC15*^23^/*GmPPC1*^22^	Glyma.12G229400	XP_006591890	12	2904	967	110864.62	6.06	Cyto
GmPEPC9	*GmPEPC7*^23^	Glyma.13G270400	XP_006593478	13	2904	967	110779.59	6.05	Cyto
*GmPEPC10*		Glyma.13G290700	XP_003543306	13	2937	978	111982.73	5.93	Cyto

Note: Chr, chromosome; ORF, open reading frame.

**Table 2:** The distribution of PEPCs in 13 different plant species.

Species	PTPC I	PTPC II	PTPC III	PTPC IV	PTPC V	BTPC I	BTPC II	Total No.
*Arabidopsis thaliana*	0	0	0	1	2	0	1	4
*Brachypodium distachyon*	3	1	1	0	0	1	0	6
*Glycine max*	0	0	0	3	4	0	3	10
*Gossypium raimondii*	0	0	0	2	3	0	1	6
*Medicago truncatula*	0	0	0	2	2	0	1	5
*Oryza sativa*	2	2	1	0	0	1	0	6
*Panicum virgatum*	3	2	2	0	0	0	1	8
*Phaseolus vulgaris*	0	0	0	2	2	0	2	6
*Ricinus communis*	0	0	0	1	1	0	0	2
*Sorghum bicolor*	2	3	0	0	0	1	0	6
*Solanum tuberosum*	0	0	0	1	4	0	0	5
*Triticum aestivum*	0	3	2	0	0	0	0	5
*Zea mays*	3	1	1	0	0	1	0	6
Total no.	13	12	7	12	18	4	10	75

**Table 3:** Conserved motifs of 75 PEPC proteins from 13 different plant species generated by MEME analysis.

Motif ID	Amino acid residues	Function of amino acid residues	MEME regular expression
Motif 1	**W**	PEP binding site	S**W**MGGDRDGNP[RN]VT[PA][EK]VT[RK]DV[CS]LL[AS]R[MW]MA[AI][ND]
Motif 2	**M D K**	PEP binding site; Mg^2+^ binding site; HCO_3_^−^ binding site	GKQEV**M**[IV]GYS**D**SG**K**DAGR[LF][ST]AAW[QF][LM]YKAQE[ED][LV][KA]
Motif 3	**R [AS]**	PEP binding site; S/A 755 site	GI[EG][ST]L**R**AIPW[IY]F[**AS**]WTQTRF[HV]LP[VA]WLG[FV]G[FV]G
Motif 4	**R**	PEP/Aspartate binding site	GG[TS]YG**R**GGGP[TC][HY]LAI[LQ]SQPP[DH][TS][IV][HNM]G[ST]LR[VS]
Motif 5	**R**	PEP binding site	[RE]F[VL]ATP[EQ][LIA]E[LIA]E[YL]G[RI][ML]NIGS**R**P[SA]
Motif 6	Unknown	Unknown	PTP[QV]DE[MA]RAG[ML][SN][YI][FV][HE][EQ][TS][IL]W[KN][GA]
Motif 7	Unknown	Unknown	VL[DG][TA]F[HR]V[IL][AS]EL[PG][SA]D[SNC][FL]GAY[IV]ISMA[TS]
Motif 8	**K**	Aspartate binding site	WPFFRVT[IL]DL[VL]EMVFA**K**G[DN]PGIAA[YL]D[KR]LLY[SA][ER]
Motif 9	Unknown	Unknown	[FL]LEPL[EL] LCY[RK]SL[CQ][SA]CGD
Motif 10	**R**	Aspartate binding site	[LI][REQ][LSIN]**R**[DLEN][SAP][YF][IL][TN][TPA][LMI]N[VM][CLF]
