# Supplementary material for: Genome-wide Analysis of Phosphoenolpyruvate Carboxylase Gene Family and Their Response to Abiotic Stresses in Soybean
Source: Sci Rep. 2016 Dec 7;6:38448. doi: 10.1038/srep38448 (PMC5141416; doi:10.1038/srep38448)
Supplement: Supplementary Information [file srep38448-s1.pdf]

Supplementary information

Genome-wide Analysis of Phosphoenolpyruvate Carboxylase Gene Family and Their Response to Abiotic Stresses in Soybean

Ning Wang, Xiujuan Zhong, Yahui Cong, Tingting Wang, Songnan Yang, Yan Li* and Junyi Gai*

National Key Laboratory of Crop Genetics and Germplasm Enhancement / National Center for Soybean Improvement / Key Laboratory for Biology and Genetic Improvement of Soybean (General, Ministry of Agriculture) / Jiangsu Collaborative Innovation Center for Modern Crop Production, Nanjing Agricultural University, Nanjing, Jiangsu, 210095, China. Correspondence and requests for materials should be addressed to Y.L. (email: yanli1@njau.edu.cn) or G.Y. (email:sri@njau.edu.cn)

*Corresponding author:

Yan Li, E-mail, yanli1@njau.edu.cn; Fax, +86-25-84399519;

Junyi Gai, E-mail, sri@njau.edu.cn; Fax, +86-25-84399519.

Supplementary information

Table S1. Primers used for qRT-PCR analyses of *GmPEPC* genes.

**Names**	**Sequences**
GmPEPC1	5’-GGCAAGTTTGTTCTAGCGGTTAGC-3’
	5’-TGGTGATAAGCAAGGCATCTCTGG-3’
GmPEPC2	5’-GTTTCAATGACACGCTCGCC- 3'
	5’-CCTTTGGCTCCAAAAGCCTC- 3'
GmPEPC3	5’-CCTGGGATTGCGGCTCTTTA-3’
	5’-CAGCAACCTGGAGGAGAAGG-3’
GmPEPC4	5’-ACTGTGGGAAGAGGAGGAGGTC-3’
	5’-CAAGTGTAGCAGCAGTGAAGCG-3’
GmPEPC5	5’-TGAGCAGGGAGAGATGGTAGAGG-3’
	5’-GGTGGGATAGGTGGACGAAGG-3’
GmPEPC6	5’-GCGTGACTGTCCAAGGTGAAG-3’
	5’-CATCCATCAAAGCCCTCCATTCC- 3'
GmPEPC7	5’-TCAAGCAAGCCAGCAGCAGAG-3’
	5’-GCATTCCAGCAGCAATACCCTTC-3’
GmPEPC8	5’-GGCTGCTCCTGCTGCTCTG-3’
	5’-TCCAATCATCACTTCTTGCTTCCC-3’
GmPEPC9	5’-TCGTGTGACAGTCCAAGGTGAAG-3’
	5’-TCCTCAGTAGCAATGACAGCCATC-3’
GmPEPC10	5’-TTGAGCTCTTACAACGCGAGTG-3’
	5’-TGATGCGGTCTCTGTACCAATC-3’
GmPPCK1	5’-CTGCTCCCGAAATCTTTGAGTCTG-3’
	5’-AAGGCTTGATGTGCGGAAATGC-3’
GmPPCK2	5’-CCGCCTCCCTCATCAAGAACC-3’
	5’-CGAACCACTCCGCCGAACC-3’
GmPPCK3	5’-ACAATCTTAAACTGGCGGACTTCG-3’
	5’-CTCCACACATCAACCTTCTCATCG-3’
GmPPCK4	5’-TCTCTCCCCTCACCCTCACATC-3’
	5’-GAGTTGCCACATCACCGAAGC-3’
GmRP15	5’-GGGAAACTGACCCACTTGAGATTGC-3’
	5’-CCACTTGCCTCTTCCAGGAGTCTTC-3’

Table S2. The nucleic acid sequences of *GmPEPC* genes.

>Glyma.01G091000 (*GmPEPC1*)
ATGACGGACACCACTGACGATATAGCGGAGGAAATCTCCTTCCAGGGCTTCGAAGAT
GACTGCAAATTGCTAGGGAACCTTCTCAACGACACTCTCCAGCGAGAGGCTGGCTCC
ACCTTCGTTGACAAGCTCGAAAAAATTCGAGTCCTTTCACAGAGTGCTTGTAATATGA
GACAAGCGGGAATGGAGGACATGGCTGAGATGCTAGAGAAGCAATTGGCTTCAGAG
TTATCCAAGATGACATTGGAAGAAGCTTTGCCCCTTGCTCGTGCATTTAGCCACCATC
TTACTTTGATGGGTATAGCTGAAACCCATCATAGGGTTCGTAAAGGAGGAAATATGG
TTCTTACTGCAAAATCTTGTGATGACATCTTTAACCACCTGTTGCAAGATGGGGTTTCT
CCAGATGAGCTTTATAACACAGTGTGCAAGCAGGAGGTTGAAATTGTTCTCACTGCTC
ATCCCACTCAAATTAACCGTCGTACCTTGCAATACAAACACCTTAAAATTGCTCATCT
TTTGGATTATAATGATCGACCTGATCTTAGCCCTGAAGATCGAGATATGTTGATTGAA
GATCTGGTGAGAGAGATAACTTCAATATGGCAGACGGATGAGCTTAGGCGTTCAAAA
CCCACACCAGTTGATGAAGCTAGGGCGGGATTGAATATTGTGGAGCAGTCACTCTGG
AAAGCTGTTCCTCATTATTTACGTCGAGTTAGCAGCGCTTTAAAGAAGCACACAGGA
AAACCACTTCCATTGACTTGCACTCCAATAAAGTTTGGATCGTGGATGGGAGGTGATA
GAGATGGAAACCCAAATGTGACAGCAAAGGTCACAAAAGATGTTTCACTTCTGTCAA
GATGGATGGCGATTGACCTCTACATTCGGGAAGTGGATGGCCTGAGATTTGAGCTAT
CCATGAACCGGTGCAGTGAAAAGTTGTCAAGACTGGCACATGAAATTCTAGAAGAAG
GTAACAATGAGGAGGATCACCATGAGCATTGGATTGAATCTATGAGTAGAAGTCAAT
CAAAACATCCTAATCAACAAGCTTCACCAATTCCAACTAAACTTCCAGCTGGTGCTCA
TTTACCCTCTTGTGCTGGGCCTGAAAAAGGTGGACCTGAGTATCCCAGACACATGCCA
GGAGCTGATCACAAGCAACCCAATCACAAGGGTGGTGAGAATTCAAGTTCAACTGAG
AGCAATGGTGGCAGTCAAAATGTCCGTTCGCCAATACCAATTTCACCAAATTCCAGTT
CTTCTTCATTAGTTTCAGTGACACGCTCTCCTTCTTTCAACTCTAGTCAACTTGTTGCT
CAGAGGAAACTGTTTGCAGAATCCCAAATAGGAAGGACCAGTTTTCAGAGGCTTTTG
GAGCCAAAGGTCCCTCAGCTTCCTGGAATTGCTCCTTATAGAGTTGTCCTTGGATATA
TAAAAGATAAGCTTCTGAGAACTCGTAGACGGTTGGAGCTTCTTATCGAGGATGGTC
CATCTGAACATGATCCCATGGATTATTATGAAACAACAGATCAGCTTTTGGAACCTCT
TCTCCTTTGTTATGAATCTTTGCAATTATGTGGATCTGGGGTGCTAGCTGATGGCCGA
CTGGCTGATCTGATTCGAAGAGTTGCTACTTTTGGCATGGTGTTAATGAAGCTTGACT
TGCGTCAGGAATCTGGCAGACATTCTGAAACAATTGATGCAATTACAAGGTATTTGG
ATATGGGTGCATACAGTGAATGGGATGAAGAAAAGAAACTGGACTTCTTAACTAGAG
AGTTGAAAGGGAAGAGACCACTGGTTCCTCCCAGTATAGAGGTTGCTCCAGATGTTA
GAGAAGTCTTGGATACGTTGCGAACTGCTGCTGAGCTAGGGAGCGATTCATTTGGTG
CCTATGTGATATCTATGGCCTCAAATGCAAGTGATGTCCTTGCAGTAGAGCTTTTGCA
GAAGGATGCACGACTTGCTGCTAGTGGAGAGTTAGGAAGAGCATGTCCTGGTGGAAC
GCTGCGGGTGGTTCCTCTATTTGAAACTGTGAAGGACCTGAGAGGAGCTGGTTCAGTT
ATCAGAAAGCTTTTGTCAATAGATTGGTACCGCCAACACATCATTAAAAACCATAAT
GGGCATCAAGAGGTTATGGTTGGGTATTCTGATTCTGGTAAAGATGCTGGGCGCTTCA
CTGCTGCTTGGGAACTTTACAAAGCTCAGGAGGATATTGTGGCTGCATGCAATGAGT
ATGGTATCAAGGTTACTCTCTTCCATGGCCGTGGAGGGAGTATTGGCCGCGGTGGTGG

CCCAACATATATGGCTATTCAGTCTCAACCACCCGGCTCTGTCATGGGGACCCTTCGA
TCAACAGAGCAAGGAGAGATGGTGCAGGCCAAGTTTGGCTTGCCACAGACAGCTGTT
AGACAGCTTGAGATATACACAACAGCTGTGCTACTTGCTACCCTTCGTCCACCTCAAC
TTCCACGTGAAGAAAAATGGCGCAATCTCATGGAAGACATTTCAAAGATCAGTTGCC
AGTGTTACCGCAATGTAGTGTATGAAAATCCAGAATTCCTGTCCTACTTCCATGAAGC
CACACCTCAATCAGAGCTTGGCTTCCTCAACATCGGTAGCCGCCCCACAAGGCGAAA
AAGCTCAACAGGAATTGGAAGCCTCCGCGCCATTCCTTGGGTGTTTGCATGGACTCAA
ACCAGGTTTGTTCTTCCTGCCTGGCTTGGAGTTGGAGCAGGTTTGAAAGGTGCTTGTG
AGAAAGGACAAACTGAAGAGCTAAAGGCGATGTACAAAGAGTGGCCCTTTTTTCAAA
GCACAATAGACTTGATTGAGATGGTTTTGGGAAAAGCAGATATTCCTATTGCTAAGC
ACTATGATGAAGTTCTTGTCTCACAGAAGAGACAAGAACTTGGAGAGCAACTGAGGA
ATGAGCTCATCACAACTGGCAAGTTTGTTCTAGCGGTTAGCGGGCACGAGAAACCTC
AGCAGAATAATAGGAGCTTAAGGAAGCTGATTGAGAGTAGACTTCCCTTTCTCAACC
CCATAAATATGTTGCAAGTGGAGATACTCAAGAGGCTAAGATGTGATGATGACAACC
TTAAAGCCAGAGATGCCTTGCTTATCACCATAAATGGTATTGCTGCAGGGATGAGGA
ACACTGGTTAA

>Glyma.02G130700 (*GmPEPC2*)

ATGACGGACATCACTGACGACATAGCGGAGGAAATCTCCTTCCAGGGCTTCGAAGAT
GACTGTAAATTGCTAGGGAACCTTCTCAACGACATTCTCCAGAGGGAGGCTGGCTCC
ACCTTCGTTGACAAGCTCGAAAAAATTCGAGTCCTTTCACAGAGTGCTTGTAATATGA
GACAAGCGGGAATGGAGGACTTGGCTGAGATGCTAGAGAAGCAATTGGCTTCAGAGT
TATCCAAGATGACGCTGGAAGAAGCTTTGCCCCTTGCTCGTGCCTTTAGCCATCATCT
TACTTTGATGGGTATAGCTGAAACCCACCATAGGGTTCGTAAAGGAGGAAATATGGT
TCTTGCTGCAAAATCTTGTGATGATATCTTTAACAACCTGTTGCAAGATGGGGTTTCT
CCAGATGAGCTTTATAACACAGTGTTCAAGCAGGAGGTTGAAATTGTTCTCACTGCTC
ATCCCACTCAAATTAACCGTCGTACCTTGCAATACAAACACCTTAAAATTGCTCATCT
TTTGGATTATAATGATCGACCTGATCTTAGCCCTGAAGATCGAGATATGTTGATTGAA
GATCTGGTGAGAGAGATAACTTCAATATGGCAGACAGATGAACTTAGGCGTTCAAAA
CCCACACCAGTAGATGAAGCTAGGGCGGGATTGAATATTGTGGAGCAGTCACTCTGG
AAAGCTGTTCCTCATTATTTACGTCGAGTTAGCAGCGCTTTAAAGAAGCACACAGGA
AAACCACTTCCATTGACTTGCACTCCAATAAAATTTGGATCTTGGATGGGAGGTGATA
GAGATGGAAACCCAAATGTGACAGCAAAGGTCACAAAAGATGTTTCACTTCTGTCAA
GATGGATGGCGATTGACCTCTACATTCGGGAAGTGGATGGCCTAAGATTTGAGCTAT
CCATGAACCAGTGCAGTGATAAGTTGTCAGAACTGGCACATGAAATTCTAAAAGAAG
GTAATGATGAGGAGGATCACCATGAGCACTGGAATGGATCTATGAGTAGAAGTCAAT
CAAAACATCCTAATCAACAAGCTTCACCACTTCCAACTAAACTTCCAGCTGGAGCTCA
TTTACCCTCTTGTGCTCGGCCTGAAGAGGGTGGATCTGAGTATCCCAGACACGTGCCA
GGAGCTGATCACAAGCAACCCAATCACAAGGGTGGTGAGACTTCAAGTTCAACTGAG
AGCAATGGTGGCAGTCAAAATGTCCGTTCGTCAATACCAATTTCACCAAATTCCAGTT
CTTCATTAGTTTCAATGACACGCTCGCCTTCCTTCAACTCTAGTCAACTTGTTGCTCAG
AGGAAACTGTTTGCAGAATCCCAAATAGGAAGGACCAGTTTTAAGAGGCTTTTGGAG
CCAAAGGTCCCTCAGGTTCCTGGAATTGCTCCTTATAGAGTTGTCCTTGGATATATAA
AAGATAAGCTTCAGAGAACTCGTAGACGGTTGGAGCTTCTTATCGAGGATGGTCCAT

CTGAACATGATCCTATGGATTATTATGAAACAACAGATCAGCTTTTAGAACCTCTTCT
CCTTTGTTATGAATCTTTGCAATTATGTGGATCTGGGGTGCTAGCTGATGGTCGACTA
GCTGATCTGATTCGAAGAGTTGCTACTTTTGGCATGGTGTTAATGAAGCTTGACTTGC
GTCAGGAATCCGGCAGACATGCTGAAACAATTGATGCAATTACGAGGTATTTGGATA
TGGGTACGTACAGTGAATGGGATGAAGAAAAGAAACTGGACTTCTTAACTAGAGAGT
TGAAAGGGAAGAGACCACTGGTTCCTCCCAGTATAGAGGTTGCTCCAGATGTTAGAG
AAGTCTTGGATACGTTCCGAACTGCTGCTGAGCTAGGGAGTGATTCATTTGGTGCCTA
TGTGATCTCTATGGCCTCAAATGCTAGTGATGTCCTTGCAGTAGAGCTTTTGCAGAAG
GATGCGCGACTTGCTGTTAGCGGGGAGTTAGGAAGAGCATGTCCTGGTGGAACGCTG
CGGGTGGTTCCTCTATTTGAAACTGTGAAGGACCTGAGAGGAGCTGGTTCAGTTATCA
GAAAGCTTTTGTCAATAGATTGGTACCGCCAACACATCATTAAAAACCATAATGGGC
ATCAAGAGGTTATGGTTGGATATTCTGATTCTGGTAAAGATGCCGGGCGCTTCACTGC
TGCTTGGGAACTTTACAAAGCTCAGGAGGATGTTGTGGCTGCATGCAATGAGTATGA
TATTAAGGTTACTCTCTTCCATGGCCGTGGAGGTAGTATTGGCCGCGGTGGTGGCCCA
ACATATATGGCTATTCAGTCTCAACCACCCGGCTCTGTCATGGGAACCCTTCGTTCAA
CTGAGCAAGGAGAGATGGTGCAGGCCAAGTTTGGCTTGCCACAGACAGCTGTCAGAC
AGCTAGAGATATACACAACAGCTGTGCTACTTGCTACCCTTCGTCCACCTCAACCGCC
ACGTGAAGAAAAATGGCGCAATCTTATGGAAGACATTTCAAAGATCAGCTGCCAATG
TTACCGCAATGTAGTGTATGAAAATCCAGAGTTCCTGTCATACTTCCAGGAAGCCACA
CCTCAATCAGAGCTTGGTTTTCTCAACATCGGCAGCCGCCCCACAAGGCGAAAGAGC
TCAACAGGAATTGGACACCTCCGTGCCATTCCTTGGGTGTTTGCATGGACTCAAACCA
GGTTTGTTCTTCCAGCCTGGCTTGGAGTTGGAGCAGGCTTGAAAGATGCTTGTGAGAA
AGGACAAACTGAAGAGCTAAAGGCCATGTACAGAGAGTGGCCTTTCTTTCAAAGCAC
AATAGACTTGATTGAGATGGTTTTGGGAAAAGCAGATATTCCTATTGCTAAGCACTAT
GATGAAGTTCTTGTCTCACAAAAGAGACAAGAACTTGGAAAGCAACTGAGGAATGAA
CTCATCTCAACTGGCAAGTTTGTTCTAGCGGTTAGTGGGCAGGAGAAACCTCAACAG
AATAATAGAAGCTTAAGGAAGCTGATTGAGAGTAGACTTCCCTTTCTGAACCCCATG
AATATGTTGCAAGTAGAGATACTCAAGAGGCTAAGATGTGATGATGACAATCTTAAA
GCCAGAGATGCCTTGCTTATCACCATAAATGGTATTGCTGCTGGGATGAGGAACACT
GGTTAA

>Glyma.06G229900 (*GmPEPC3*)

ATGGCAAACAGGAACTTGGAAAAGATGGCATCCATCGATGCTCAGCTTCGGCTGTTG
GTTCCAGCCAAAGTGAGTGAGGATGATAAACTGGTTGAGTATGATGCTTTGCTCTTGG
ATCGATTCCTTGATATTCTTCAGGATTTACATGGGGAGGATCTGAAAGAAACGGTTCA
AGAGGTGTATGAGCTTTCTGCTGAGTATGAAGGGAAACATGACCCAAAGAAACTGGA
GGAACTTGGAAATCTGATAACTAGTTTGGATGCTGGAGATTCCATTGTAGTTGCCAAG
TCCTTTTCCCACATGCTTAACTTGGCCAACTTAGCCGAAGAGGTCCAAATTGCACACA
GCCGAAGGAACAAGTTGAAGAAAGGTGATTTTGCTGATGAAAACAATGCCACCACTG
AATCAGATATTGAAGAAACTCTCAAGAAACTTGTTGGGGATATGAAGAAGTCTCCTC
AGGAAGTTTTTAATGCACTGAAAAACCAGACTGTTGATCTGGTTCTTACTGCTCATCC
AACTCAATCTGTCCGTAGGTCTTTGCTTCAAAAACATGGAAGGATAAGAAATAATTTA
ACTCAGTTGTATGCCAAAGACATTACTCCTGATGATAAGCAGGAACTTGATGAGGCT
CTACAGAGGGAGATCCAAGCTGCATTTCGTACTGATGAAATCAGGAGGACCCCTCCA

ACCCCACAAGATGAGATGAGAGCAGGGATGAGTTACTTCCATGAAACAATTTGGAAG
GGTGTACCAACATTTCTACGTCGTGTTGATACAGCTTTGAAGAATATAGGGATCAATG
AGCGTGTCCCTTATAATGCTCCTCTCATTCAATTTTCCTCTTGGATGGGTGGAGATCGT
GATGGCAATCCAAGAGTAACTCCAGAAGTGACAAGAGATGTTTGCTTATTGGCTAGA
ATGATGGCTGCTAACTTGTACTACTCCCAGATAGAGAATCTTATGTTTGAACTGTCAA
TGTGGCGCTGCAATGATGAGCTACGTGTTCGTGCAGATGAACTTAACAGGTCTTCCAA
GAAAAATTCAGTGGCAAAACACTACATAGAATTCTGGAAAGTCATTCCTCCAAATGA
ACCATATCGTGTGCTACTGGGTGAAGTAAGGAATAGGCTTTACCATACTCGAGAGCG
CTCGCGCCATTTGCTAGCTCATGGCTACTCTGACATTCCAGAGGAAGAAACATTCACC
AATGTTGAGGAGTTCTTGGAACCCCTTGAGCTCTGTTACAGATCACTCTGTGCTTGTG
GCGATCGAGCAATTGCTGATGGAAGCCTTCTAGATTTCTTGAGACAAGTCTCTACTTT
TGGACTCTCCCTAGTGAGGCTCGACATAAGGCAAGAGTCAGACCGCCACACCGATGT
CTTAGATGCCATCACCAAGCATTTGGAAATAGGCTCATACCAGGAATGGTCCGAGGA
AAAACGGCAGCAATGGCTTTTATCCGAGTTGAGTGGCAAAAGGCCCCTATTTGGCCC
TGACCTTCCCCAAACAGAAGAAATCAGAGACGTTTTGGACACATTCCATGTCATAGC
AGAGCTTCCACCAGACAACTTTGGAGCATACATCATCTCAATGGCAACTGCACCTTCT
GATGTGCTTGCAGTTGAGCTTCTGCAGCGAGAATGCCATGTCAAGCATCCACTAAGA
GTTGTGCCATTGTTTGAGAAGCTAGCTGATCTGGAAGCAGCACCAGCTGCATTGGCAC
GGTTGTTCTCCGTAGACTGGTACAGAAACAGGATCAATGGGAAGCAGGAAGTGATGA
TTGGCTATTCTGATTCTGGCAAAGATGCTGGAAGGTTTTCGGCCGCGTGGCAGCTGTA
TAAGGCTCAAGAGGAGCTTATAAAGGTGGCTAAAGAGTATGGTGTGAAGCTGACAAT
GTTCCATGGCCGTGGAGGGACGGTCGGAAGAGGAGGTGGTCCAACTCACCTTGCTAT
TCTGTCTCAGCCTCCGGATACCATTCATGGATCGCTGCGCGTGACTGTCCAAGGTGAA
GTTATTGAGCAATCATTTGGAGAGCAGCACTTGTGCTTCAGAACGCTTCAAAGGTTCA
CTGCAGCTACTCTAGAACATGGAATGCATCCTCCAATTTCTCCTAAACCAGAATGGCG
GGCTTTGATGGATGAGATGGCTGTCATTGCCACTGAGGAGTACCGGTCCATTGTGTTC
AAAGAACCACGATTTGTTGAGTATTTCCGCCTGGCCACACCTGAGTTGGAATATGGA
AGGATGAACATTGGAAGTCGACCGGCAAAGAGGAGGCCAAGTGGAGGTATTGAGAC
ACTGCGCGCCATACCTTGGATCTTTGCATGGACACAAACAAGGTTCCATCTTCCAGTG
TGGCTAGGCTTTGGTGCAGCATTCGAACATGTTATTGAGAAGGATGTTAGGAATATTC
ATGTGCTGCAGGAGATGTATAATCAATGGCCTTTCTTTAGGGTCACTATTGATTTAGT
GGAAATGGTGTTTGCCAAAGGAGACCCGGGGATCGCTGCTCTTTATGATAGGCTCCTT
GTTTCAGAGGATCTGTGGTCATTTGGAGAGCAGTTGAGGACCAAGTATGAAGAAACC
AAGGAATTCCTCCTTCAGGTGGCTGGCCATAGGGATCTTCTTGAAGGAGATCCATACT
TGAAGCAAAGACTGCGCTTGCGTGATTCTTATATTACTACCCTAAACGTGTGCCAAGC
CTACACATTGAAACGTATACGCGATCCAAACTATAACGTGAAGCTGCGCCCTCACAT
CTCCAAGGAGTCTATAGAGGTAAGTAAACCTGCTGATGAACTCATAACACTTAACCC
AACAAGTGAATATGCACCTGGTTTGGAAGACACCCTCATTCTCACCATGAAGGGTATT
GCTGCTGGCTTGCAAAACACTGGCTAA
>Glyma.06G277500 (*GmPEPC4*)
ATGGGTACACGGAACTTTGAGAAAATGGCTTCTATTGATGCTCAGTTGAGGTTGCTAG
CACCAAGTAAGGTTTCTGATGATGACAAGCTTGTTGAATATGATGCTTTGTTGCTGGA
CCGTTTCCTTGACATTCTTCAGGATTTGCATGGCGATGATATTAGAGAAACGGTTCAA

GACTGTTATGAGCTGTCTGCTGAGTATGAAGGGCAGAATAATCCTCAGAAGTTGGAG
GAACTTGGGAACATGCTAACTGGTCTTGATGCTGGGGATTCTATTGTGATTTCCAAGT
CGTTTGCTCATATGCTTAATTTGGCAAATTTGGCAGAAGAAGTTCAGATTGCCTACCG
TAGAAGGATTAAGTTATTGAAGAAGGGTGATTTTGCTGATGAGAATTCTGCTATCACT
GAGTCAGATATTGAAGAGACTTTCAAGAGGCTTGTGAATCAACTGAAGAAGACACCT
CAAGAAATCTTTGATGCTTTGAAGAGTCAAACTGTAGATTTGGTCTTAACTGCTCATC
CTACTCAGTCTGTTCGTAGATCTTTGCTGCAAAAGCATGGCAGGATAAGGAATTGTTT
GACACAGTTGTATGCTAAAGACATAACACCGGACGATAAACAGGAGCTTGATGAGGC
TTTACAAAGAGAGATTCAAGCTGCATTTCGTACGGATGAAATTCGAAGGACCCCTCCT
ACACCACAAGATGAGATGAGAGCAGGAATGAGCTACTTTCACGAGACGATCTGGAA
AGGCATACCAAAATTTTTGCGCCGGGTTGACACTGCTCTGAAGAACATTGGCATAAA
TGAACGTGTTCCATATAATGCCCCTGTTATTCAATTCTCTTCTTGGATGGGAGGAGAT
CGTGATGGTAACCCCAGGGTAACTCCAGAAGTTACAAGGGATGTGTGTTTGCTGGCT
AGAATGATGGCTGCTAATTTGTACTTCTCTCAGATTGAGGATCTCATGTTTGAGTTGT
CTATGTGGCGCTGCAATGATGAGCTACGTGTTCGTTCTGATGAACTCCTTAGCTCCTC
AAAGAGAGATGCAAAACATTATATTGAGTTTTGGAAACAGATTCCTCCCAATGAGCC
GTATCGTGTTATTCTCGGTGATGTAAGGGACAAATTATATAATACACGTGAACGTGCT
CGGCAGTTGTTAGCCAATGGAAGCTCTGAAATCCCTGAGGAGACTACCTTCACAAAT
GTTGAACAGTTCCTGGAGCCTCTTGAATTATGTTATAGATCGCTGTGTGCATGTGGTG
ACCAACCAATAGCTGATGGAAGCCTTCTTGATTTCTTGCGCCAAGTTTCCACATTTGG
GCTTTCACTTGTAAGACTTGATATCCGTCAAGAATCAGATCGGCACACTGATGTTATG
GATGCTATTACAAACCACTTAGAGATTGGATCTTATCGAGAGTGGTCTGAGGAACGC
AGGCAGGAATGGCTGCTATCCGAGCTCAGTGGAAAGCGCCCTCTCTTTGGTCCTGATC
TTCCTAAAACAGAAGAAATTGCTGATGTATTGGAAACCTTCCATGTCATTGCGGAACT
TCCTTCAGACAGCTTTGGTGCCTATATCATCTCAATGGCAACAGCTCCATCTGATGTG
CTTTCTGTAGAACTTTTACAACGTGAATGTCATGTGAAGCAGCCATTAAGGGTTGTTC
CATTGTTTGAAAAACTTGCTGATCTTGAGGCTGCTCCTGCTGCGGTAGCCCGCCTATT
CTCTATAGATTGGTACAGAGACCGTATCAATGGGAAACAAGAAGTTATGATAGGGTA
CTCGGACTCGGGAAAAGATGCTGGTCGTTTCTCTGCAGCATGGGCACTATACAAGGC
TCAAGAAGAGCTCATAAAGGTTGCAAAAGAGTTTGGTGTTAAGCTCACAATGTTCCA
TGGCAGAGGAGGAACTGTGGGAAGAGGAGGAGGTCCCACTCATCTTGCTATATTATC
TCAGCCACCGGATACTATTCATGGCTCACTTCGGGTAACAGTGCAAGGTGAAGTTATT
GAACAGTCATTTGGAGAGGAGCACTTGTGCTTCAGAACACTTCAGCGCTTCACTGCTG
CTACACTTGAGCATGGAATGCACCCTCCTGTGGCACCAAAACCAGAGTGGCGTGCCC
TCATGGATGAGATGGCTGTCATTGCTACAGAGGAGTATCGCTCCATTGTTTTCCAGGA
ACCCCGTTTCGTTGAGTACTTCCGATGTGCTACACCTGAGCTGGAGTATGGAAGAATG
AACATTGGCAGTCGTCCGTCAAAACGAAAGCCAAGTGGAGGAATTGAATCACTTCGT
GCAATCCCTTGGATCTTTGCTTGGACTCAGACTAGGTTTCATCTACCTGTATGGCTTGG
CTTTGGGGCAGCATTTAGCCATGTTATTAAGAAGGATCCAAAGAATCTCCAAATGCTT
CAAGATATGTATAATCAATGGCCTTTCTTCAGGGTCAGCCTTGACTTGGTTGAGATGG
TGTTCGCCAAGGGAGACCCTGGGATTGCGGCTCTTTATGACAAACTCCTAGTGTCAGA
GGAACTGTGGCCATTTGGCGAGCGATTAAGGTCTATGTTTGAAGAAACCAAGAGCCT
TCTCCTCCAGGTTGCTGGACACAAAGATCTCCTTGAAGGAGACCCCTACTTGAAGCAA

AGACTTCGACTTCGCGATTCATACATCACAACCCTCAATGTCCTGCAAGCCTACACAT
TGAAGCGAATTCGTGATCCTGACTACCATGTGAAGTTGAGGCCACATTTGTCCAAAG
ACTACATGGAATCAAACAAGCCAGCAGCTGAGCTTGTGAAACTCAACCCTACAAGTG
ATTATGCTCCTGGTCTGGAGGATACCCTTATTTTGACAATGAAGGGTATTGCTGCTGG
CATGCAAAACACTGGTTAA
>Glyma.10G205500 (*GmPEPC5*)
ATGACTGACATCACTGATGATATTGCTGAGGAAATCTCCTTCCAGAGCTTCGATGATG
ACTGCAGGTTGCTTGGTAATCTCCTCAATGACATTCTCCAGCGTGAAGTTGGCACCAA
CTTGCTTGACAAGATCGAAAGGACTCGAGTCCTTGCTCAGAGTGGTTGTAATATGAG
GCAGGCGGGTATTGTAAACATGGCAGAGATGCTTGAGAAGCAGTTGGCTTCGGAGTT
ATCAAAGATGACACTAGAAGAAGCTTTCACCCTTGCTCGTGCCTTCAGCCATTATCTT
ACTTTGATGGGTATAGCTGAGACCCACCATAGGGTTCGTAAAGGAGGGAATATGGCA
CAAATTGCAAAATCTTGCGATGATATATTTAACCAGCTGGTGCAGGGTGGAGTCCCCC
CAGAAGAACTTTATGACACAGTCTGCAAGCAGGAGGTTGAAATTGTTCTCACTGCTC
ATCCCACACAGATTAACCGTCGAACCTTACAGTTTAAACACATTAGAATTGCTCATCT
TTTGGATTACAATGATCGACCTGATCTTAGCACTGAAGATCGAGAAATGGTGATTGA
AGATCTGGTGAGAGAGATAACTTCAATATGGCAGACAGATGAGCTTAGGCGCCAGAA
ACCCACTCCAGTTGATGAAGCTAGAGCTGGTTTCAATATTGTGGAGCAGTCACTCTGG
AAAGCTGTCCCTCATTATTTACGTCGTGTCAGCAATGCATTAAAGAAGCATACAGGA
AAGCCACTTCCTTTGACTTGCACTCCCATAAAGTTTGGATCTTGGATGGGAGGTGATA
GAGATGGAAACCCAAATGTGACAGCAAAGGTCACAAAAGATGTTTCACTTCTATCTA
GATGGATGGCGATTGACCTCTATATTCGGGAAGTGGATAGCCTCAGATTTGAGCTATC
CATGAACCAGTGCAGTGATAGGTTGTCAAGATTGGCACATGAAATTCTAGAAGCTAA
GCATGAGAATCGCCGTGAGAATTGGAATCAGTCTGCGAATAGAAGTCTCACACTTCC
AACACAACTTCCAGCTAGAGCTCATTTACCTTCTATTGCTGAAAATGGTGAATCTCGG
CATCCCAGACTAGACATTCCAGCACCTGATTACATGCAATCCAATCACAAGGATGGT
GGGGTTTCTGTAAGTTCAACTACATCAAAACTTGCCAATCCCAATACTCGATTACCAG
GAACAAGTTCAGCAAATTCCAGTGCTTCTTCAGCTGCACTTGGTCAAAAGAAATTGTA
TGCAGAATCCCAGACAGGAAAGTCCACTTTTCAAAAGCTTTTGGAGCCAATGCTTCCT
CAACTTCCTGGAATTGCTCCTTATAGAATTGTCCTGGGGAATGTTAAGGATAAGCTTG
AGAAAAGTCGTAGACGGTTAGAAATTCTTCTTGAGGATGTTGCATGTGACTATGATCC
TTTGGATTACTATGAAACATCTGATCAGCTTTTGGAACCTCTGCTCCTCTGTTATGAAT
CTCTGCAATCGTGTGGATCTGGGGTGCTAGCTGATGGTCGACTTGCTGATCTGATTCG
TAGAGTTGCTACCTTTGGAATGGTGTTAATGAAGCTTGACTTGCGTCAGGAATCTGGG
AGACATGCAGAAGCACTTGATGCAATAACACAGTACTTAGATATGGGTACTTACAGT
GAATGGGATGAAGAAAAGAAGTTGGACTTCTTAACAAGAGAACTTAAAGGGAAGAG
GCCTCTTGTTCCTGTTAGTATAGAGGTTCATCCTGATGTTAAAGAAGTCTTGGATACA
TTCCGAATTGCCGCTGAACTGGGGAGTGATTCACTTGGAGCTTATGTGATCTCTATGG
CCTCAAATGCAAGTGATGTCCTTGCAGTAGAGCTTTTACAGAAGGATGCACGGCTTGC
TGCTATTGGGGAGTTGGGAAAAGCATGTCCTGGTGGAACGTTGCGGGTTGTCCCTCTG
TTTGAAACTGTGAAAGACCTGAGAGGAGCTGGTTCAGTTATCCGGAAACTTTTATCAA
TAGACTGGTACCATGAACACATCATTAAGAACCATAATGGACATCAAGAGGTTATGG
TTGGATATTCTGATTCTGGTAAAGATGCTGGTCGCTTTACTGCTGCTTGGGAACTTTAC

AAAGCTCAGGAGGATGTTGTAGCTGCTTGCAATGATTATGGAATAAAGGTTACTCTAT
TCCATGGTCGTGGAGGCAGTATTGGTCGTGGTGGCCCAACATATCTGGCTATTCA
GTCCCAACCCCCTGGCTCTGTGATGGGAACGCTTCGGTCTACTGAGCAGGGAGAGAT
GGTAGAGGCTAAGTTTGGGTTGCCACAGATAGCTGTTAGACAACTTGAGATATACAC
AACAGCTGTACTACTTGCAACCCTTCGTCCACCTATCCCACCCCGAGAAGAAAAATG
GCGTAATGTCATGGAAGAGATCTCAAACATCAGTTGTCAGTGTTACCGCAATGTAGT
GTATGAAAATCCAGAATTCCTGGCCTACTTCCATGAAGCCACACCAGAGGCAGAACT
TGGCTTCCTTAACATAGGTAGCCGCCCTACAAGAAGGAAGAGCTCAGTAGGAATCGG
ACACCTTCGTGCAATTCCCTGGTTATTTGCATGGACACAAACAAGATTCGTTCTTCCA
GCTTGGCTTGGAGTCGGAGCAGGTTTAAAAGGAGCTTGCGAGAAAGGTTACACCGAA
GAGCTAAAAGCCATGTACAAAGAATGGCCCTTCTTTCAAAGTACCATAGATCTTATTG
AGATGGTTTTGGGGAAAGCTGACATTCCTATAGCCAAGCACTATGATGAAGTCCTTGT
GTCAAAGGAGAGGCAAGAGCTTGGCCATGAACTAAGAAGTGAGCTCATGACAGCTG
AAAAGTTTGTCATGGTTATTAGTGGGCACGAGAAACTTCAGCAGAATAATAGGAGCT
TGAGGAGGCTAATTGAGAATAGACTTCCCTTCCTTAATCCCTTGAACATGTTGCAGGT
GGAGATACTCAAGAGGTTAAGACGTGATGATGACAACCGTAAGATCAGAGATGCTTT
GCTTATCACCATAAATGGGATTGCTGCAGGGATGAAGAATACAGGTTGA
>Glyma.12G161300 (*GmPEPC6*)
ATGGCGAACAGGAACTTGGAAAAGATGGCATCGATTGATGCTCAGCTTCGGCTGTTG
GTTCCGGCCAAAGTGAGTGAGGATGACAAACTGGTTGAGTATGATGCTTTGCTTTTGG
ATCGGTTCCTTGATATTCTTCAGGATTTACATGGGGAGGATCTGAAAGAAACGGTTCA
AGAGGTGTATGAGCTTTCTGCTGAGTATGAAGGTAAGCATGACCCAAAGAAACTAGA
GGAACTTGGAAATCTGATAACTAGTTTGGATGCTGGAGATTCCATTGTGGTTGCCAAG
TCCTTTTCCCACATGCTTAACTTGGCCAACTTAGCCGAAGAGGTCCAAATTGCCCACA
GTCGAAGGAACAAGTTGAAGAAAGGTGATTTTGCTGATGAAAACAATGCCACCACTG
AATCAGACATTGAAGAAACTCTAAAGAAACTTGTGGTGGATATGAAGAAGTCTCCTC
AGGAAGTTTTTGATGCACTTAAAAACCAGACTGTTGATCTGGTTCTTACTGCTCATCC
TACTCAATCTGTCCGTAGGTCTTTGCTTCAAAAACATGGAAGGATAAGAAATAATTTA
ACTCAGTTGTATGCCAAAGACATCACTCCTGATGATAAGCAGGAACTTGACGAGGCT
CTACAGAGGGAGATCCAAGCTGCATTCCGTACTGATGAAATCAGGAGGACCCCTCCA
ACCCCACAAGATGAGATGAGAGCAGGGATGAGCTACTTCCATGAAACAATTTGGAAG
GGTGTACCCACATTTCTACGTCGTGTTGATACAGCTTTGAAGAATATAGGGATCAACG
AACGTGTCCCTTATAATGCTCCTCTCATTCAATTTTCTTCTTGGATGGGTGGAGATCGT
GATGGCAATCCAAGAGTAACTCCTGAAGTGACCAGAGATGTTTGCTTATTGGCTAGA
ATGATGGCTGCTAACTTGTACTACTCCCAGATAGAGGATCTTATGTTTGAGCTGTCAA
TGTGGCGCTGCAATGACGAGCTACGTGTCCGTGCAGATGAACTTAACAGGTCTTCCA
AGAAAAATTCAGTCGCAAAACACTACATAGAATTTTGGAAAGCCATTCCTCCAAATG
AACCATATCGTGTGCTACTGGGTGAAGTAAGGAATAGGCTTTACCAGACTCGTGAAC
GCTCACGCCATTTGCTAGCTCACGGATACTCTGACATTCCAGAGGAAGAGACTTTTAC
CAATGTTGAGGAGTTCTTGGAACCCCTTGAACTCTGTTACAGATCACTCTGTGCTTGT
GGCGATCGTGCAATTGCCGATGGAAGCCTTCTAGATTTCTTGAGACAAGTCTCTACTT
TCGGACTCTCCCTAGTGAGGCTTGACATAAGGCAAGAGTCAGACCGCCACACAGACG
TCTTAGACGCCATCACCAAACATTTAGAAATAGGCTCATACCAGGAATGGTCGGAGG

AAAAACGGCAGCAATGGCTTTTATCTGAGTTGAGTGGCAAACGGCCCCTATTCGGCC
CTGATCTTCCCCAAACCGAAGAAATCAGAGACGTGTTGGAGACATTCCATGTCATAG
CAGAGCTTCCACTAGACAACTTTGGAGCATACATCATCTCAATGGCAACTGCACCTTC
TGATGTGCTTGCAGTTGAGCTTCTGCAGCGCGAATGCCATGTCAAGCATCCACTAAGA
GTTGTGCCATTGTTTGAGAAGCTAGCTGATCTAGAAGCAGCACCGGCCGCGTTGGCG
CGATTGTTCTCTGTAGACTGGTACAGAAACAGGATCAATGGGAAGCAGGAAGTGATG
ATAGGCTATTCTGATTCTGGCAAAGATGCTGGAAGGTTTTCAGCTGCATGGCAGCTGT
ATAAGGCTCAGGAGGAGCTTATAATGGTGGCTAAGCAGTATGGTGTGAAGCTGACAA
TGTTCCATGGTCGTGGAGGGACAGTTGGAAGAGGAGGTGGTCCAACTCACCTTGCTA
TTCTGTCTCAGCCTCCTGAAACCATTCATGGATCGCTGCGCGTGACTGTCCAAGGTGA
AGTTATTGAGCAATCGTTTGGAGAGCAGCACTTGTGCTTCAGAACGCTTCAAAGGTTC
ACTGCAGCTACTCTAGAACATGGAATGCACCCTCCAATTTCTCCTAAACCGGAATGGA
GGGCTTTGATGGATGAGATGGCTGTCATTGCCACTGAGGAGTACCGGTCCATTGTGTT
CAAAGAACCACGATTTGTTGAGTATTTCCGCCTGGCCACACCTGAGTTGGAGTACGG
GAGGATGAACATTGGAAGTCGACCAGCAAAGAGGAGGCCAAGTGGAGGTATTGAGA
CACTCCGTGCCATACCTTGGATCTTTGCCTGGACACAAACAAGGTTCCATCTTCCAGT
GTGGCTAGGCTTTGGTGCAGCATTCAAACATGTTATTGAGAAGGATGTTAGGAATATT
CATGTGCTGCAGGAGATGTACAATCAATGGCCTTTCTTTAGGGTCACTATTGATTTAG
TGGAAATGGTGTTTGCCAAAGGAGACCCAGGGATAGCTGCTCTTTATGATAGGCTCCT
TGTTTCAGAGGATCTGTGGTCATTTGGGGAGCAGTTGAGGACCATGTACGAAGAAAC
CAAGGAACTCCTCCTTCAGGTGGCTGGCCATAGGGATCTTCTTGAAGGAGATCCATAC
TTGAAGCAAAGACTGCGCTTGCGTGATTCTTATATTACTACCCTAAACGTGTGCCAAG
CCTACACGTTGAAACGTATCCGTGATCCAAACTATAATGTGAAGCTGCGCCCTCACAT
CTCCAAAGAGTCTATAGAGATAAGTAAACCTGCTGATGAACTTATAACACTTAACCC
AACAAGTGAATATGCACCTGGTTTGGAAGACACCCTCATTCTCACCATGAAGGGTATT
GCTGCTGGCTTGCAAAACACTGGCTAA
>Glyma.12G210600 (*GmPEPC7*)
ATGGCTGCTCGTAACATCGAGAAGATGGCCTCAATTGATGCTCAACTGAGGTTGCTG
GCGCCACGCAAGGTGTCTGATGATGACAAACTCGTTGAGTATGATGCCTTGTTGTTGG
ATCGTTTCCTTGACATTCTTCAGGATTTGCATGGTGAAGATATCAGACAAACGGTCCA
AGATTGTTACGAGCTGTCAGCTGAGTACGAAGGAGAGCATAAGCCTGAAAAGTTGGA
GGAACTTGGGAATATGCTGACGGGTCTTGATGCTGGGGATTCAATTGTTATTGCCAAG
TCATTTTCTCACATGCTCAATTTGGCCAACTTGGCAGAAGAAGTTCAAATTGCGTATC
GAAGAAGGATCAAGTTACTGAAGAAGGGTGATTTTGCTGATGAGAACTCTGCCATTA
CTGAGTCAGACATTGAAGAGACCTTCAAGAAGCTTGTGGCTCAACTGAAGAAGACAC
CCCAGGAAATCTTTGATGCTTTGAAGAACCAAACTGTGGATTTGGTTCTAACTGCTCA
TCCTACTCAGTCTGTTCGCAGATCTTTGCTGCAAAAGCATGGAAGGATAAGGAATTGT
TTGACACAATTATATGCTAAGGACATAACACCAGATGATAAGCAGGAACTTGATGAG
GCTTTACAAAGAGAGATTCAAGCTGCATTTCGCACAGATGAAATTCGAAGGACTCCT
CCTACCCCACAAGATGAGATGAGGGCAGGAATGAGCTACTTTCATGAGACAATTTGG
AAAGGTGTACCACAGTTTCTGCGTCGGGTAGATACAGCTCTGAAGAACATTGGAATT
AATGAACGTGTCCCATATAATGCTCCTGTTATTCAGTTCTCTTCTTGGATGGGAGGAG
ACCGTGATGGTAATCCTAGAGTAACCCCTGAAGTTACAAGGGATGTGTGTTTGCTGGC

TAGAATGATGGCTGCTAATATGTACTTCTCTCAGATAGAGGATCTCATGTTTGAGTTG
TCTATGTGGCGTTGCACTGACGAGCTACGTGTTCGTGCTCATGAACTCCATAGGTCCT
CAAAGAGAGATGCAAAACATTATATTGAGTTTTGGAAACAGATTCCTCCAAATGAGC
CATATCGTGTTATTCTTGGTGATGTCAGAGACAAACTATATAACATTCGGGAACGTGC
TCGCCATTTATTAGCCAATGGGACATCTGATATCCCTGAGGAGACAACCTTCACTAAC
GTTGAGCAGTTTCTGGAGCCCCTTGAACTATGCTACAGGTCACTCTGTGCATGTGGTG
ACAGACCAATAGCAGATGGAAGCCTCCTTGATTTCCTGCGGCAAGTTTCCACATTTGG
ACTCTCACTAGTGAGACTTGACATCCGTCAAGAGTCAGATAGGCACACTGATGTTATG
GATGCAATCACAAAACACTTAGACATTGGATCATACCGAGAGTGGCCCGAGGAAAAG
AGGCAGGAGTGGCTCTTGTCTGAACTCAGTGGAAAGCGCCCTCTCTTTGGCCATGACC
TTCCCAAAACAGAAGAAATCACCGATGTTTTGGAAACATTCCGTGTCATTTCAGAGCT
TCCCTCAGACAACTTCGGTGCCTACATCATATCAATGGCAACATCCCCATCTGATGTG
CTTGCTGTTGAGCTTTTACAACGTGAATGCCATGTGAAGCAGCCACTAAGGGTGGTGC
CACTGTTTGAAAAGCTTGCTGATCTTGAGGCCGCTCCGGCTGCGGTGGCACGTCTTTT
CTCTATAGATTGGTACAGAAACCGCATTGATGGGAAGCAAGAAGTTATGATAGGGTA
CTCAGACTCAGGAAAAGATGCAGGCCGTCTTTCTGCGGCTTGGGCGCTTTACAAGGCT
CAAGAAGAACTTGTGAAGGTTGCTAAGGAGTATGGTGTTAAACTTACAATGTTTCAT
GGGAGAGGAGGGACTGTTGGAAGAGGAGGAGGTCCAACTCATCTTGCTATATTGTCT
CAGCCACCAGACACCATTCATGGCTCACTTCGGGTAACGGTTCAGGGTGAAGTCATT
GAGCAGTCTTTTGGAGAGGAACACTTGTGCTTTAGAACACTTCAGCGCTTCACTGCAG
CTACACTTGAGCATGGCATGCATCCTCCTGTGTCACCGAAACCCGAATGGCGTGCCCT
CATGGATGAAATGGCTGTCATTGCAACAAAGGAGTATCGCTCTGTTGTTTTCAAAGAA
CCTCGTTTTGTTGAATATTTCAGATGTGCAACTCCTGAGTTGGAGTATGGAAGAATGA
ACATTGGCAGTCGTCCATCAAAGCGAAAGCCAAGTGGAGGAATTGAATCACTACGTG
CTATTCCTTGGATTTTTGCATGGACACAAACGAGGTTTCATTTGCCAGTGTGGCTTGG
TTTTGGGTCAGCATTTAAGCATGTAGTTGAGAAAGATCCGAAGAATCTCCAAATGCTT
CAGGACATGTACAATCAATGGCCTTTCTTCAGGGTCACCCTGGACTTGGTTGAGATGG
TGTTTGCTAAGGGAGACCCGGGGATTGCAGCCCTATTTGACAAACTCCTAGTATCAGA
AGAGCTGCGTCCATTTGGAGAAAATTTAAGAGCTAAATACGAAGAAACCAAGAGCTT
TCTCCTCCAGGTTGCTGGGCACAAGGATATTCTTGAAGGAGACCCCTACTTGAAGCAA
AGACTTCGTCTTCGTGACTCATACATCACAACCCTCAATGTGTTGCAAGCTTACACAT
TGAAGCGAATTCGTGATCCTGACTACCATGTGAAGTTGAGGCCACACTTGTCAAAGG
ACTACATGGAATCAAGCAAGCCAGCAGCAGAGCTTGTTAAACTTAACCCCAAAAGCG
AGTATGCTCCTGGTCTTGAGGACACCCTTATTTTGACAATGAAGGGTATTGCTGCTGG
AATGCAAAATACCGGTTAA
>Glyma.12G229400 (*GmPEPC8*)
ATGGCGACCAGGAACTTGGAAAAGATGGCATCCATTGATGCACAGCTTAGGCAATTG
GCTCCTGCCAAAGTGAGTGAGGATGACAAACTGATTGAGTATGATGCTCTTCTGCTGG
ATCGGTTCCTTGATATCCTTCAAGATTTACATGGGGAGGATCTGAAGGAAACAGTTCA
AGAAGTGTATGAACTTTCAGCTGAGTATGAAGGAAAGCATGACCCTAAGAAACTGGA
AGAACTTGGAAATTTGATAACCAGTTTGGATGCTGGGGACTCTATTCTTGTTGCCAAG
TCCTTTTCCCACATGCTTAATTTGGCCAACTTGGCTGAAGAGGTCCAGATTTCTCGCC
GCCGAAGAAACAAGTTGAAGAAAGGGGATTTTGCAGATGAGAACAATGCAACTACA

GAATCAGACATTGAAGAAACTCTCAAGAAACTTGTATTTGACTTGAAGAAGTCTCCTC
AGGAAGTTTTTGATGCACTGAAAAACCAGACTGTTGATTTGGTTCTTACTGCTCATCC
TACTCAATCAATTCGTAGATCTTTGCTTCAAAAGCATGGAAGGATAAGGAATTGTTTA
TCTCAATTGTATGCCAAAGACATTACTCCTGATGATAAGCAGGAGCTTGATGAGGCTC
TACAAAGGGAGATTCAAGCTGCCTTCCGTACAGATGAAATCAGGAGGACCCCTCCAA
CACCACAAGATGAGATGAGAGCAGGGATGAGCTACTTCCATGAAACAATTTGGAACG
GTGTTCCCAGATTTCTGCGCCGTGTAGACACAGCTTTGAACAATATCGGGATTAAAGA
GCGTGTTCCTTATAATGCTCCCCTTATTCAATTTTCTTCTTGGATGGGGGGTGATCGCG
ATGGTAATCCAAGAGTAACTCCTGAAGTGACAAGGGATGTTTGCTTATTGGCTAGAA
TGATGGCTGCTAATTTGTATTATTCCCAGATAGAAGATCTTATGTTTGAGCTCTCTATG
TGGCGCTGCAATGATGAACTACGCGTTCGTGCAGAAGAACTTCACAGGTCTTCCAAG
AAAGATGAAGTTGCAAAACACTATATAGAATTTTGGAAAAAGGTTCCCCCAAATGAA
CCATATCGTGTGGTACTCGGTGAAGTAAGGGATAGGCTCTATCAGACTCGTGAGCGTT
CTCGCCATTTGCTTTCTAATGGGTACTCTGACATTCCAGAGGAAGCCACTTTCACCAA
TGTTGAGGAGTTCCTGGAATCTCTTGAACTATGTTACAGATCACTATGTGCTTGTGGT
GATAGAGCAATTGCTGATGGAAGCCTTCTTGATTTCATGAGACAAGTCTCCACTTTTG
GACTGTCACTAGTGAGGCTTGATATCAGGCAAGAGTCAGATCGTCACACTGATGTGC
TGGATGCCATTACCAAACACTTGGAAATTGGCTCGTACCAGGAATGGTCTGAAGAGA
AAAGACAGGAATGGTTGTTGTCTGAGTTAAGTGGCAAAAGGCCTCTATTTGGACCTG
ACCTTCCTCAAACTGAAGAAATTAGAGATGTTTTGGACACATTTCATGTCATAGCAGA
ACTACCACCAGACAACTTTGGAGCCTATATCATATCAATGGCAACTGCACCATCTGAT
GTGCTTGCAGTTGAGCTTCTACAACGTGAATGTCACATCAAGCATCCCTTAAGAGTTG
TGCCATTGTTTGAGAAGCTAGCTGATCTAGAGGCTGCTCCTGCTGCTCTGGCACGGTT
GTTCTCGATAGACTGGTACAGAAATAGGATCAATGGGAAGCAAGAAGTGATGATTGG
ATACTCAGATTCAGGGAAAGATGCTGGGAGGTTCTCTGCAGCATGGCAGCTATATAA
GGCTCAGGAGGAACTTATAAATGTTGCCAAGAAATTTGGTGTTAAGCTAACCATGTTC
CATGGTCGCGGTGGAACTGTTGGAAGAGGAGGTGGACCTACTCATCTTGCTATTCTGT
CTCAACCTCCAGACACAATCCATGGATCACTTCGTGTGACAGTCCAAGGTGAAGTCAT
TGAGCAATCATTTGGAGAACAACACTTGTGCTTTAGAACACTACAACGTTTCACTGCC
GCCACTCTAGAACATGGCATGCACCCCCCAATTTCGCCAAAACCAGAATGGCGTGCT
TTGATGGATCAGATGGCTGTCATTGCTACTGAGGAATACCGTTCCATTGTATTCAAGG
AACCACGCTTTGTTGAGTATTTCCGCCTGGCTACACCAGAGTTGGAGTATGGAAGGAT
GAATATTGGAAGTCGACCAGCAAAGAGAAGACCTAGTGGAGGCATTGAAACACTGC
GTGCAATACCTTGGATTTTTGCATGGACTCAGACAAGGTTTCATCTTCCAGTGTGGCT
AGGCTTTGGAGCAGCATTTAAAAAAGTCATTGAGGAAAATGTTAAGAATCTCAATAT
GCTGCAAGAGATGTACAATCAATGGCCTTTCTTTAGGGTCACACTTGATTTGGTGGAA
ATGGTGTTTGCCAAAGGAGATCCGAAAATTGCCGCTCTGAATGATAGACTCCTTGTTT
CAAAGGATCTGTGGCCGTTTGGGGATCAATTGAGGAACAAATATGAAGAAACTAGGA
AACTCCTACTTCAGGTGGCTGGACACAAGGAAATTCTTGAAGGGGACCCTTACTTGA
AGCAAAGACTCAGGCTTCGTCATGCTCCCATTACCACCCTCAATATTGTCCAAGCTTA
CACATTGAAACGTATCCGTGATCCTAACTACAATGTGAAGGTGCGCCCCCGCATATCA
AAGGAATCTGCAGAGGCAAGCAAATCAGCTGATGAACTTGTCAAATTGAACCCAACA
AGTGAATATGCCCCTGGTTTGGAAGACACACTCATTCTCACTATGAAGGGTATTGCTG

CTGGCATGCAGAACACTGGTTAA
>Glyma.13G270400 (*GmPEPC9*)
ATGGCGAACAGGAACCTGGAAAAGATGGCATCCATCGATGCACAGCTTAGGCAATTG
GCTCCTGCCAAAGTGAGTGAGGATGACAAACTGATTGAGTATGATGCTCTTCTGTTGG
ATCGGTTCCTTGATATTCTTCAAGATTTACATGGGGAGGATCTGAAGGAAACAGTCCA
AGAAGTATATGAACTTTCAGCTGAGTATGAAGGAAAGCATGACCCTAAGAAACTGGA
AGAACTTGGAAATTTGATAACCAGTTTGGATGCTGGGGACTCTATTCTTGTTGCTAAG
TCCTTTTCCCACATGCTTAATTTGGCCAACTTGGCTGAAGAGGTCCAGATTTCTCGCC
GCCGAAGAAACAAGTTGAAGAAAGGGGATTTTGCAGATGAGAACAACGCAACTACA
GAATCAGACATTGAAGAAACTCTCAAAAAACTTGTATTTGGCTTGAAGAAGTCTCCTC
AGGAAGTTTTTGATGCACTGAAAAACCAGACTGTTGATTTGGTTCTTACTGCTCACCC
TACTCAATCAATTCGTAGATCTTTGCTTCAAAAGCATGGAAGGATAAGGAATTGTTTA
TCTCAATTGTATGCCAAAGACATCACTCCTGATGATAAGCAGGAGCTTGATGAGGCTC
TACAAAGGGAGATTCAAGCTGCCTTCCGTACAGATGAAATCAGGAGGACCCCTCCAA
CACCACAAGATGAGATGAGAGCAGGGATGAGCTACTTCCATGAAACAATTTGGAATG
GTGTTCCCAGATTTCTGCGCCGTGTAGACACAGCTTTGAACAATATAGGGATTAAAGA
GCGTGTTCCTTATAATGCTCCCCTTATTCAATTTTCTTCTTGGATGGGTGGTGATCGGG
ATGGTAATCCAAGAGTAACTCCTGAAGTGACAAGGGATGTTTGCTTATTGGCTAGAA
TGATGGCTGCTAATTTGTATTATTCCCAGATAGAAGATCTTATGTTTGAGCTCTCTATG
TGGCGCTGCAATGATGAACTACGCGTTCGTGCAGAAGAACTTCACAGGTCTTCCAAG
AAAGATGAAGTTGCAAAGCACTATATAGAATTCTGGAAAAAGGTTCCCCCAAATGAA
CCATATCGTGTGGTACTTGGTGAAGTAAGGGATAGGCTCTATCAAACTCGCGAGCGTT
CTCGCCATTTGCTTTCTAATGGGTACTCTGACATTCCAGAGGAAGCCACTTTCACCAA
TGTTGAGGAGTTCCTAGAATCTCTTGAACTATGTTATAGATCACTATGTGCTTGTGGT
GATAGAGCAATTGCTGATGGAAGCCTTCTTGATTTCATGAGACAAGTCTCTACTTTTG
GATTGTCACTAGTGAGGCTTGATATCAGGCAAGAGTCAGATCGTCACACTGATGTGCT
GGATGCCATTACCAAACACTTGGAAATTGGCTCATACCAGGAATGGTCTGAAGAGAA
AAGACAGGAATGGTTGTTGTCTGAGTTAAGTGGCAAAAGGCCTCTGTTTGGACCTGA
CCTTCCCCAAACCGAAGAAATTAGAGATGTTTTGGACACATTTCATGTCATAGCAGAA
CTACCACCAGACAACTTTGGAGCCTATATCATATCAATGGCAACTGCACCATCTGATG
TGCTTGCAGTTGAGCTTCTACAACGTGAATGCCACATCAAGCATCCCTTAAGAGTTGT
GCCATTGTTTGAGAAGCTAGCTGATCTAGAGGCTGCTCCTGCTGCTTTGGCACGGTTG
TTCTCGATAGACTGGTATAGAAATAGGATCAATGGGAAGCAAGAAGTGATGATTGGA
TACTCAGATTCAGGAAAAGATGCTGGGAGGTTCTCTGCAGCATGGCAGCTATATAAG
GCTCAGGAGGAACTTATAAATGTTGCCAAGAAATTTGGAATTAAGCTAACCATGTTC
CATGGTCGCGGTGGAACTGTTGGAAGAGGAGGTGGACCTACTCATCTTGCTATTCTGT
CTCAACCTCCAGACACAATCCATGGATCACTTCGTGTGACAGTCCAAGGTGAAGTCAT
TGAGCAATCATTTGGAGAACAACACTTGTGTTTTAGAACACTACAACGTTTCACTGCT
GCCACTCTAGAACATGGCATGCACCCCCCGATTTCTCCAAAACCAGAATGGCGTGCTT
TGATGGATCAGATGGCTGTCATTGCTACTGAGGAATACCGTTCCATTGTATTCAAGGA
ACCACGCTTTGTTGAGTATTTCCGCCTGGCTACACCGGAGTTGGAGTATGGAAGGATG
AACATTGGAAGTCGACCAGCAAAGAGAAAGCCTAGTGGAGGCATTGAAACACTGCG
TGCAATACCTTGGATTTTTGCATGGACTCAGACAAGGTTTCATCTTCCAGTGTGGCTA

GGCTTTGGAGCAGCATTTAAAGAAGTCATTGAGAAAAATGTTAACAATCTCAATATG
CTGCAAGAGATGTACAATCAATGGCCTTTCTTTAGGGTCACACTTGATTTGGTGGAAA
TGGTGTTTGCCAAAGGAGATCCGAAAATTGCTGGTCTGAATGATAGGCTCCTTGTTTC
AAAGGATCTGTGGCTGTTTGGGGATCAATTGAGGAACAAATATGAAGAAACTAAGAA
ACTCCTACTTCAGGTGGCTGGACACAAGGAAATTCTTGAAGGGGACCCTTACTTGAA
GCAAAGACTCAGGCTTCGTCATGCTCCCATTACCACCCTCAATATTGTCCAAGCTTAC
ACATTGAAACGTATCCGTGATCCTAACTACAATGTGAAGGTGCGCCCCCGCATATCA
AAGGAATCTGCAGAGGCAAGCAAATCAGCTGATGAACTTATAAAACTGAACCCAACA
AGTGAATATGCTCCTGGTTTAGAAGACACCCTCATTCTCACTATGAAGGGTATTGCTG
CTGGCATGCAGAACACTGGTTAA
>Glyma.13G290700 (*GmPEPC10*)
ATGGCTGCTCGTAACATCGAGAAGATGGCCTCAATTGATGCTCAGTTGAGGTTGCTGG
CACCACGCAAGGTGTCTGATGATGACAAACTCGTTGAGTATGATGCTTTGTTGTTGGA
CCGCTTCCTTGACATTCTTCAGGATTTGCATGGTGAAGATATCAGACAAACGGTCCAA
GATTGTTATGAGCTGTCAGCTGAGTACGAAGGAGAGCATAAGCCTGATAAGTTGGAG
GAACTTGGGAATATGCTGACTGGTCTTGATGCTGGGGATTCAATTGTTATTGCCAAGT
CATTTTCTCACATGCTCAATTTGGCCAACTTGGCAGAAGAAGTTCAAATTGCGTACCG
AAGAAGGATCAAGTTGCTGAAGAAGGGTGATTTTGCTGATGAGAACTCTGCCATTAC
TGAGTCAGACATTGAAGAGACCTTCAAGAAGCTTGTGGCTCAACTGAAGAAGACACC
CCAGGAAATCTTTGATGCTTTGAAGAACCAAACTGTGGATTTGGTCCTAACTGCTCAT
CCTACTCAGTCTGTTCGCAGATCGTTGCTGCAAAAGCATGGAAGACTAATTATTTCTG
TTGGTGATCATTCTCTTCAGGATAAAGGAATTTGTTTGACACAATTATATGCTAAGGA
CATTACACCAGATGATAAGCAGGAACTTGATGAGGCTTTACAAAGAGAGATTCAAGC
TGCATTTCGCACGGATGAAATTCGAAGGACTCCTCCTACCCCACAAGATGAGATGAG
GGCAGGAATGAGCTACTTTCATGAGACAATTTGGAAAGGTGTACCACAGTTTCTGCG
TCGGGTAGATACAGCTCTGAAGAACATTGGAATTAATGAACGTGTCCCATATAATGC
TCCTGTTATTCAGTTCTCTTCTTGGATGGGAGGAGATCGTGATGGTAATCCTAGAGTA
ACCCCTGAAGTTACCAGGGATGTGTGTTTGCTGGCTAGAATGATGGCTGCTAATGTGT
ACTTCTCTCAGATAGAAGATCTCATGTTTGAGTTGTCTATGTGGCGTTGCACTGACGA
GCTCCGTGATCGTGCTCATGAACTCCATAGGTCCTCAAAGAGAGATGCAAAACATTA
CATTGAGTTTTGGAAACAGGTTCCTCCAAATGAGCCATATCGTGTTATTCTTGGTGAT
GTCCGAGACAAACTGTATAACACACGGGAACGTGCTCGCCAATTATTAGCCAATGGG
ACCTCTGATATCCCCGAGGAGACAACTTTCACTTACGTTGAGCAGTTCCTGGAGCCTC
TTGAACAATGCTATAGATCACTCTGTGCCTGTGGTGACAGACCTATAGCAGATGGAA
GCCTCCTTGATTTCCTGCGGCAAGTTTCGACATTTGGACTCTCACTTGTGAGACTTGAC
ATCCGTCAAGAGTCAGATAGGCACACTGATGTCATGGATGCAATCACAAAACACTTA
GAGATCGGATCATACCGAGAGTGGCCCGAGGAAAAGAGGCAGGAGTGGCTCTTATCT
GAACTACGTGGAAAACGCCCTCTCTTTGGCCATGACCTTCCCAAAACAGAAGAAATC
AATGATGTTTTGGAAACCTTCCATGTCATTTCAGAACTTCCCTCAGACAACTTCGGTG
CCTACATCATATCAATGGCAACAGCCCCATCTGATGTGCTTGCTGTTGAGCTCTTACA
ACGCGAGTGCCATGTGAAGCAGCCTCTAAGGGTGGTGCCACTGTTTGAAAAGCTTGC
TGATCTTGAGGCTGCTCCGGCTGCTGTGGCGCGGCTTTTCTCGATAGATTGGTACAGA
GACCGCATCAATGGGAAGCAAGAAGTTATGATAGGGTACTCAGACTCGGGAAAAGA

TGCTGGCCGTCTTTCTGCGGCTTGGGCGCTTTACAAGGCTCAAGAAGAACTTGTGAAG
GTTGCTAAGGAGTATGGTGTTAAACTTACAATGTTTCATGGGAGGGGAGGGACTGTT
GGAAGAGGAGGAGGTCCAACTCATCTTGCTATATTGTCTCAGCCACCAGACACCATC
CATGGCTCACTTCGGGTGACGGTTCAGGGTGAAGTCATTGAGCAGTCTTTTGGAGAG
GAGCACTTGTGCTTTAGGACACTTCAGAGGTTCACTGCAGCTACACTTGAGCATGGAA
TGCATCCTCCTGTGTCACCGAAACCAGAATGGCGCGCCCTCATGGACGAGATGGCTG
TCATTGCAACAAAGGAGTATCGCTCTGTTGTTTTCAAAGAGCCTCGTTTTGTTGAATA
TTTCAGATGTGCAACTCCTGAGTTGGAGTATGGAAGAATGAACATTGGCAGTCGTCC
ATCAAAGCGAAAGCCAAGTGGAGGAATTGAATCACTACGTGCTATTCCTTGGATTTTT
GCATGGACACAAACGAGGTTTCATTTGCCAGTGTGGCTTGGTTTTGGGTCAGCATTTA
AGCATGTAGTTGAGAAAGATCCAAAGAATCTCCAAATGCTTCAGGACATGTACAATC
AATGGCCTTTCTTCAGGGTCACCCTTGACTTGGTTGAGATGGTGTTCGCCAAGGGAGA
CCCCGGGATTGCATCCCTATTTGACAAACTCCTAGTATCAGAAGAGCTGCGTCCATTT
GGAGAGAATTTAAGATCTAAATTCGAAGAAACAAAGAGGTTTCTCCTCCAGGTTGCT
GGGCACAAGGATATTCTTGAAGGAGACCCCTACTTGAAGCAAAGACTTCGTCTTCGT
GACTCATACATCACAACCCTCAATGTGTTGCAAGCCTACACATTGAAGCGGATCCGTG
ATCCTGACTACCATGTGAAGTTGAGGCCACACTTGTCAAAGGACTACATGGAATCAA
GCAAGCCAGCAGCAGAGCTTGTTAAACTTAACCCCAAAAGCGAGTATGCTCCTGGTC
TTGAGGACACCCTTATTTTGACAATGAAGGGTATTGCTGCTGGCATGCAAAATACAG
GTTAA

Table S3. The information of *PEPC* genes in other 12 plant species.

Species	Gene name	Gene ID	Species	Gene name	Gene ID
Arabidopsis thaliana	AtPEPC1	AT1G53310	*Phaseolus vulgaris*	PvPEPC1	Phvul.003G024800
	AtPEPC2	AT2G42600		PvPEPC2	Phvul.005G066400
	AtPEPC3	AT3G14940		PvPEPC3	Phvul.005G095300
	AtPEPC4	AT1G68750		PvPEPC4	Phvul.007G101300
Brachypodium distachyon	BdPEPC1	Bradi1g39167		PvPEPC5	Phvul.011G130400
	BdPEPC2	Bradi2g00910		PvPEPC6	Phvul.011G160200
	BdPEPC3	Bradi2g06620	*Ricinus communis*	RcPEPC1	29645.m000074
	BdPEPC4	Bradi2g50380		RcPEPC2	29983.m003317
	BdPEPC5	Bradi3g09210	*Sorghum bicolor*	SbPEPC1	Sobic.002G167000
	BdPEPC6	Bradi4g27910		SbPEPC2	Sobic.003G100600
Gossypium raimondii	GrPEPC1	Gorai.002G061400		SbPEPC3	Sobic.003G301800
	GrPEPC2	Gorai.004G272100		SbPEPC4	Sobic.004G106900
	GrPEPC3	Gorai.006G001300		SbPEPC5	Sobic.007G106500
	GrPEPC4	Gorai.008G095300		SbPEPC6	Sobic.010G160700
	GrPEPC5	Gorai.008G187900	*Solanum tuberosum*	StPEPC1	PGSC0003DMT400032245
	GrPEPC6	Gorai.013G173500		StPEPC2	PGSC0003DMT400039808
Medicago truncatula	MtPEPC1	Medtr0002s0890		StPEPC3	PGSC0003DMT400039809
	MtPEPC2	Medtr2g076670		StPEPC4	PGSC0003DMT400052608
	MtPEPC3	Medtr2g092930		StPEPC5	PGSC0003DMT400054791
	MtPEPC4	Medtr4g079860	*Triticum aestivum*	TaPEPC1	Traes_3AL_681758236
	MtPEPC5	Medtr8g463920		TaPEPC2	Traes_3DL_A62754CCA
Oryza sativa	Osppc-b	LOC_Os01g02050		TaPEPC3	Traes_5AL_65FC8CBE2
	Osppc4	LOC_Os01g11054		TaPEPC4	Traes_5BL_6642E2A8B
	Osppc3	LOC_Os01g55350		TaPEPC5	Traes_5DL_DA19E2C89
	Osppc1	LOC_Os02g14770	*Zea mays*	ZmPEPC1	GRMZM2G069542_T01
	Osppc2a	LOC_Os08g27840		ZmPEPC2	GRMZM2G074122_T01
	Osppc2b	LOC_Os09g14670		ZmPEPC3	GRMZM2G082780_T01
Panicum virgatum	PviPEPC1	Pavir.Aa02787		ZmPEPC4	GRMZM2G083841_T01
	PviPEPC2	Pavir.Ab00954		ZmPEPC5	GRMZM2G110714_T01
	PviPEPC3	Pavir.Eb00827		ZmPEPC6	GRMZM2G473001_T01
	PviPEPC4	Pavir.Eb03218			
	PviPEPC5	Pavir.J09522			
	PviPEPC6	Pavir.J27884			
	PviPEPC7	Pavir.J34423			
	PviPEPC8	Pavir.J38540			

Table S4. ***Cis*-elements in the promoter regions of *GmPEPC* genes in soybean.**

Cis-elements	*GmPEPC1*	*GmPEPC2*	*GmPEPC3*	*GmPEPC4*	*GmPEPC5*	*GmPEPC6*	*GmPEPC7*	*GmPEPC8*	*GmPEPC9*	*GmPEPC10*
AAAC-motif		1					1			
ABRE			1		1	1	2	3	4	
ACE				1		1		2		1
AE-box	1	2	1			2		2	1	2
ARE		2	1	1	1	3			3	2
as-2-box								1	1	
AT1-motif					1	2	1		1	
ATCT-motif	1		2	3			1			
ATC-motif									1	
AuxRR-core								1		
Box I	1		2	1	1	4	2		1	
Box III					1					
Box 4	6	4		2	2	2	4	5	7	4
Box-W1	1	1			2					
CAT-box			1							
CATT-motif		1				1				
CCAAT-box	1	2				1				
CCGTCC-box					1					
CGTCA-motif	1	1	3			3			1	
chs-CMA2a							1			
circadian		3	2			1	1	2	2	1
E2Fb	1									
ELI-box3									1	
EIRE								1		
ERE	1					2	2		1	
F-box	1									
G-box		2	2	1	1	2	2	3	3	5
GA-motif	1	1			1			1	1	
GAG-motif	1	1	2	1		2	2	2		4
Gap-box						1				
GARE-motif	0	1			1		1	1		
GATA-motif	1			1	1		2			
GCC-box						1				
GC-motif		1								
GCN4_motif					1	2	1			
GT1-motif	1	2	1					3		1
HSE	1	2	1		3		1			
I-box				1	1		2	1		
LAMP-element			1							
LTR	1					1				
MBS	2	2	1		2	2	3	1	1	2
MRE	1	2		1			1	2	2	
O_2-site			1		1	1		2	1	
P-box			2		1					
Pc-CMA2a								1	1	
rbcS-CMA7a		1								
S-box		1	1							
Skn-1_motif	1	4	4	2	1	5	3	4	7	2
Sp1		2	1		1					
TC-rich repeats	0	1		1	2	1		1	1	1
TCA-element			1		2	2	1	1		1
TCCC-motif		1								
TCT-motif									1	
TGA-element	1				2			1		2
TGACG-motif	1	1	3			3			1	
TGG-motif		1								
W-box	1	1			2					
WUN-motif						1				

Figure S1. Evolutionary relationships (A) and gene structures (B) of *PEPC* from soybean and other 12 plant species. (A) The phylogenetic tree was constructed with MEGA 6.0 using the neighbor-joining (NJ) method with 1,000 bootstrap replicates based on a multiple alignment of 75 *PEPC* genes. Bootstrap values higher than 50% are shown on the nodes. (B) Exon/intron structures of *PEPC* genes from 13 different plant species. Exons and introns are represented by green boxes and black lines, respectively.

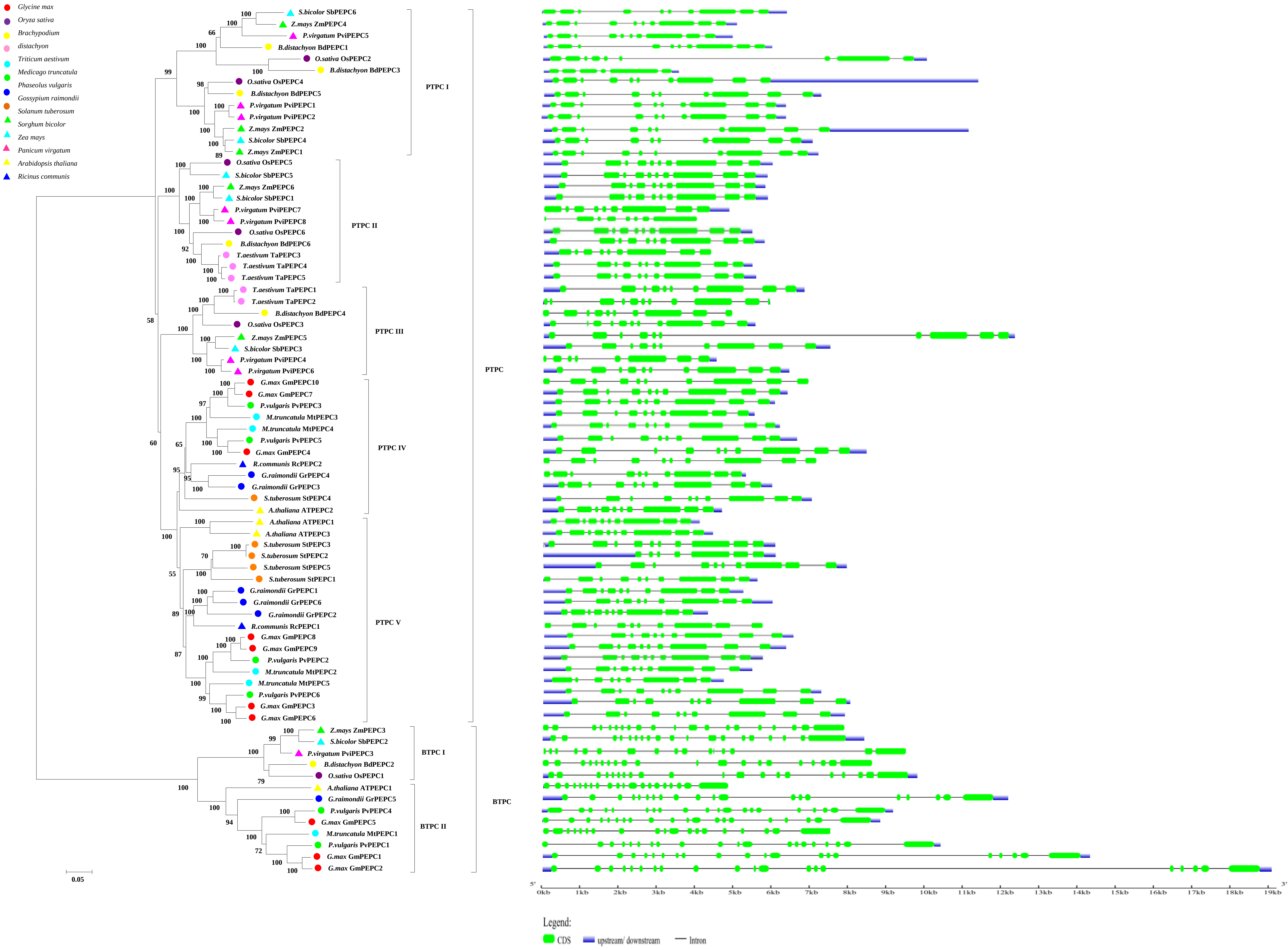

Glycine max
Oryza sativa
Brachypodium
distachyon
Triticum aestivum
Medicago truncatula
Phaseolus vulgaris
Gossypium raimondii
Solanum tuberosum
Sorghum bicolor
Zea mays
Panicum virgatum
Arabidopsis thaliana
Ricinus communis
S.bicolor SbPEPC6
Z.mays ZmPEPC4
P.virgatum PviPEPC5
B.distachyon BdPEPC1
O.sativa OsPEPC2
B.distachyon BdPEPC3
O.sativa OsPEPC4
B.distachyon BdPEPC5
P.virgatum PviPEPC1
P.virgatum PviPEPC2
Z.mays ZmPEPC2
S.bicolor SbPEPC4
Z.mays ZmPEPC1
O.sativa OsPEPC5
S.bicolor SbPEPC5
Z.mays ZmPEPC6
S.bicolor SbPEPC1
P.virgatum PviPEPC7
P.virgatum PviPEPC8
O.sativa OsPEPC6
B.distachyon BdPEPC6
T.aestivum TaPEPC3
T.aestivum TaPEPC4
T.aestivum TaPEPC5
T.aestivum TaPEPC1
T.aestivum TaPEPC2
B.distachyon BdPEPC4
O.sativa OsPEPC3
Z.mays ZmPEPC5
S.bicolor SbPEPC3
P.virgatum PviPEPC4
P.virgatum PviPEPC6
G.max GmPEPC10
G.max GmPEPC7
P.vulgaris PvPEPC3
M.truncatula MtPEPC3
M.truncatula MtPEPC4
P.vulgaris PvPEPC5
G.max GmPEPC4
R.communis RcPEPC2
G.raimondii GrPEPC4
G.raimondii GrPEPC3
S.tuberosum StPEPC4
A.thaliana ATPEPC2
A.thaliana ATPEPC1
A.thaliana ATPEPC3
S.tuberosum StPEPC3
S.tuberosum StPEPC2
S.tuberosum StPEPC5
S.tuberosum StPEPC1
G.raimondii GrPEPC1
G.raimondii GrPEPC6
G.raimondii GrPEPC2
R.communis RcPEPC1
G.max GmPEPC8
G.max GmPEPC9
P.vulgaris PvPEPC2
M.truncatula MtPEPC2
M.truncatula MtPEPC5
P.vulgaris PvPEPC6
G.max GmPEPC3
G.max GmPEPC6
Z.mays ZmPEPC3
S.bicolor SbPEPC2
P.virgatum PviPEPC3
B.distachyon BdPEPC2
O.sativa OsPEPC1
A.thaliana ATPEPC1
G.raimondii GrPEPC5
P.vulgaris PvPEPC4
G.max GmPEPC5
M.truncatula MtPEPC1
P.vulgaris PvPEPC1
G.max GmPEPC1
G.max GmPEPC2
PTPC I
PTPC II
PTPC III
PTPC IV
PTPC V
PTPC
BTPC I
BTPC II
BTPC
0.05
5'
3'
0kb
1kb
2kb
3kb
4kb
5kb
6kb
7kb
8kb
9kb
10kb
11kb
12kb
13kb
14kb
15kb
16kb
17kb
18kb
19kb
Legend:
CDS
upstream/ downstream
Intron

Figure S2. Conserved motifs of PEPC proteins. All motifs were identified by MEME with the complete amino acid sequences of 75 PEPCs from 13 different plant species. Lengths of motifs were displayed proportionally. The classification of PEPCs was indicated according to the phylogenetic relationship on the left.

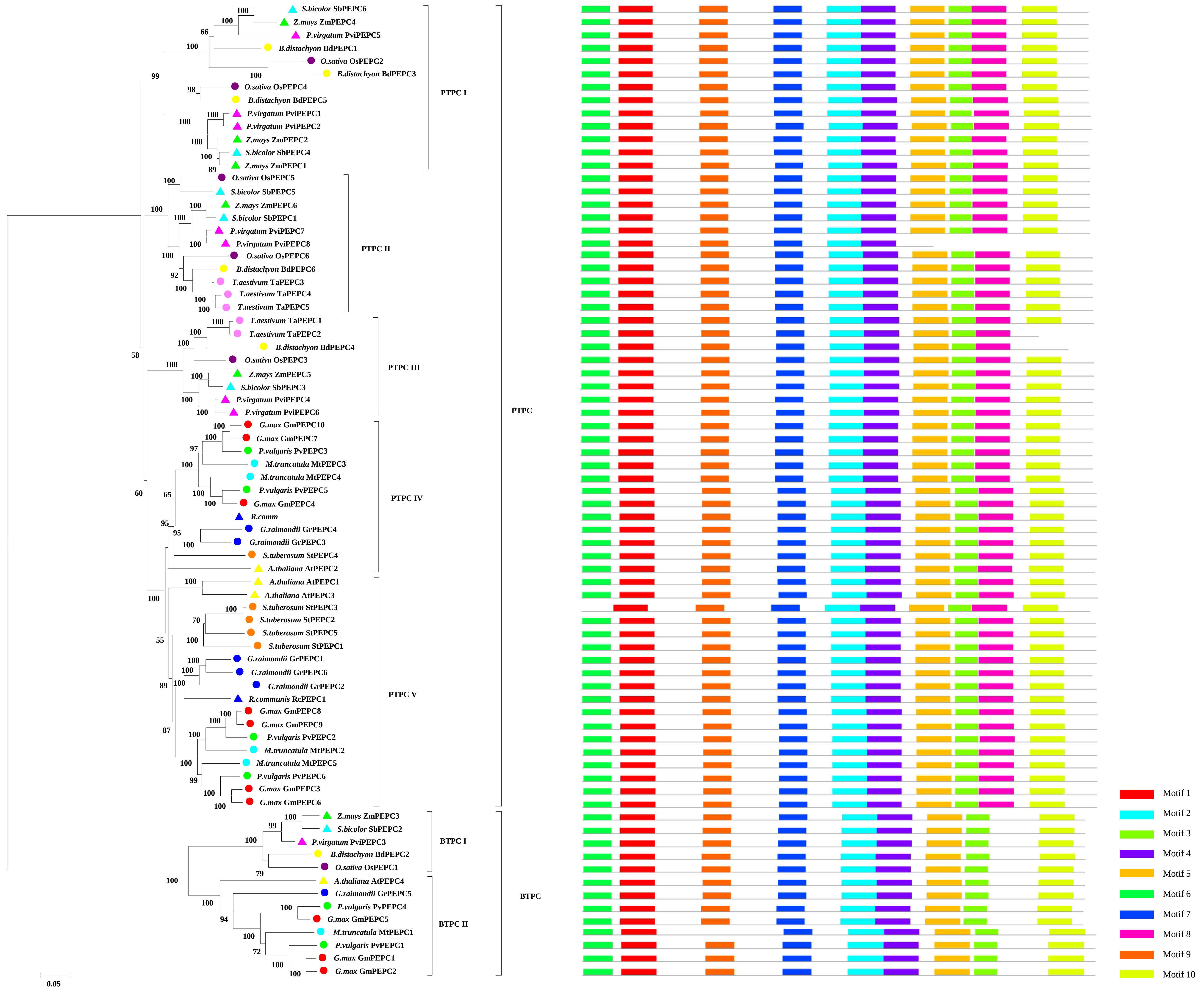

S.bicolor SbPEPC6
Z.mays ZmPEPC4
P.virgatum PviPEPC5
B.distachyon BdPEPC1
O.sativa OsPEPC2
B.distachyon BdPEPC3
O.sativa OsPEPC4
B.distachyon BdPEPC5
P.virgatum PviPEPC1
P.virgatum PviPEPC2
Z.mays ZmPEPC2
S.bicolor SbPEPC4
Z.mays ZmPEPC1
O.sativa OsPEPC5
S.bicolor SbPEPC5
Z.mays ZmPEPC6
S.bicolor SbPEPC1
P.virgatum PviPEPC7
P.virgatum PviPEPC8
O.sativa OsPEPC6
B.distachyon BdPEPC6
T.aestivum TaPEPC3
T.aestivum TaPEPC4
T.aestivum TaPEPC5
T.aestivum TaPEPC1
T.aestivum TaPEPC2
B.distachyon BdPEPC4
O.sativa OsPEPC3
Z.mays ZmPEPC5
S.bicolor SbPEPC3
P.virgatum PviPEPC4
P.virgatum PviPEPC6
G.max GmPEPC10
G.max GmPEPC7
P.vulgaris PvPEPC3
M.truncatula MtPEPC3
M.truncatula MtPEPC4
P.vulgaris PvPEPC5
G.max GmPEPC4
R.comm
G.raimondii GrPEPC4
G.raimondii GrPEPC3
S.tuberosum StPEPC4
A.thaliana AtPEPC2
A.thaliana AtPEPC1
A.thaliana AtPEPC3
S.tuberosum StPEPC3
S.tuberosum StPEPC2
S.tuberosum StPEPC5
S.tuberosum StPEPC1
G.raimondii GrPEPC1
G.raimondii GrPEPC6
G.raimondii GrPEPC2
R.communis RcPEPC1
G.max GmPEPC8
G.max GmPEPC9
P.vulgaris PvPEPC2
M.truncatula MtPEPC2
M.truncatula MtPEPC5
P.vulgaris PvPEPC6
G.max GmPEPC3
G.max GmPEPC6
Z.mays ZmPEPC3
S.bicolor SbPEPC2
P.virgatum PviPEPC3
B.distachyon BdPEPC2
O.sativa OsPEPC1
A.thaliana AtPEPC4
G.raimondii GrPEPC5
P.vulgaris PvPEPC4
G.max GmPEPC5
M.truncatula MtPEPC1
P.vulgaris PvPEPC1
G.max GmPEPC1
G.max GmPEPC2
PTPC I
PTPC II
PTPC III
PTPC IV
PTPC V
PTPC
BTPC I
BTPC II
BTPC
0.05
Motif 1
Motif 2
Motif 3
Motif 4
Motif 5
Motif 6
Motif 7
Motif 8
Motif 9
Motif 10

Figure S3. Patterns of *PEPC* genes expression in soybean leaves under different stresses and hormone treatments. The 14-d soybean seedlings were treated with 100 µM ABA (abscisic acid), 100 µM ACC (1-aminocyclopropane-1-carboxylic acid), 25 µM $AlCl_3$ (pH 4.3), 4 ℃ cold, 100 µM GA (gibberellins), 100 µM JA (jasmonic acid), 200 mM NaCl, and 20 % (w/v) PEG (polyethylene glycol) 6000. The relative expression levels were analyzed by qRT-PCR using the house-keeping gene *GmRP15* as the internal control. Values are means ± SD of three biological replicates. Statistical significance of differences between control and treated groups was analyzed using Student's t-test (* indicates $P < 0.05$).

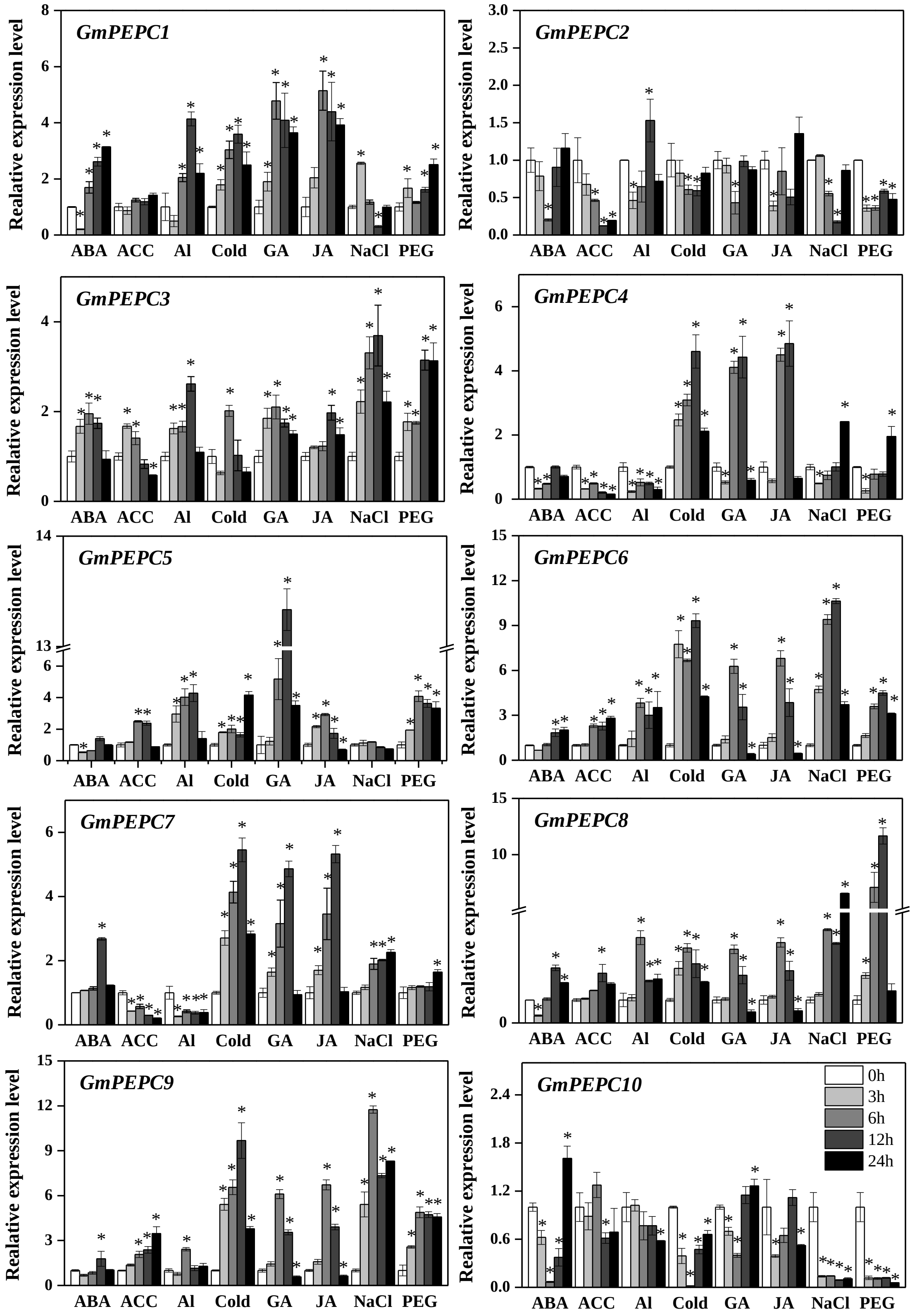
GmPEPC1
GmPEPC2
GmPEPC3
GmPEPC4
GmPEPC5
GmPEPC6
GmPEPC7
GmPEPC8
GmPEPC9
GmPEPC10
Realative expression level
ABA ACC Al Cold GA JA NaCl PEG
0h
3h
6h
12h
24h

Figure S4. Patterns of *PEPC* genes expression in soybean roots under different stresses and hormone treatments. The 14-d soybean seedlings were treated with 100 μM ABA (abscisic acid), 100 μM ACC (1-aminocyclopropane-1-carboxylic acid), 25 μM $AlCl_3$ (pH 4.3), 4 ℃ cold, 100 μM GA (gibberellins), 100 μM JA (jasmonic acid), 200 mM NaCl, and 20 % (w/v) PEG (polyethylene glycol) 6000. The relative expression levels were analyzed by qRT-PCR using the house-keeping gene *GmRP15* as the internal control. Values are means ± SD of three biological replicates. Statistical significance of differences between control and treated groups was analyzed using Student's t-test (* indicates $P < 0.05$).

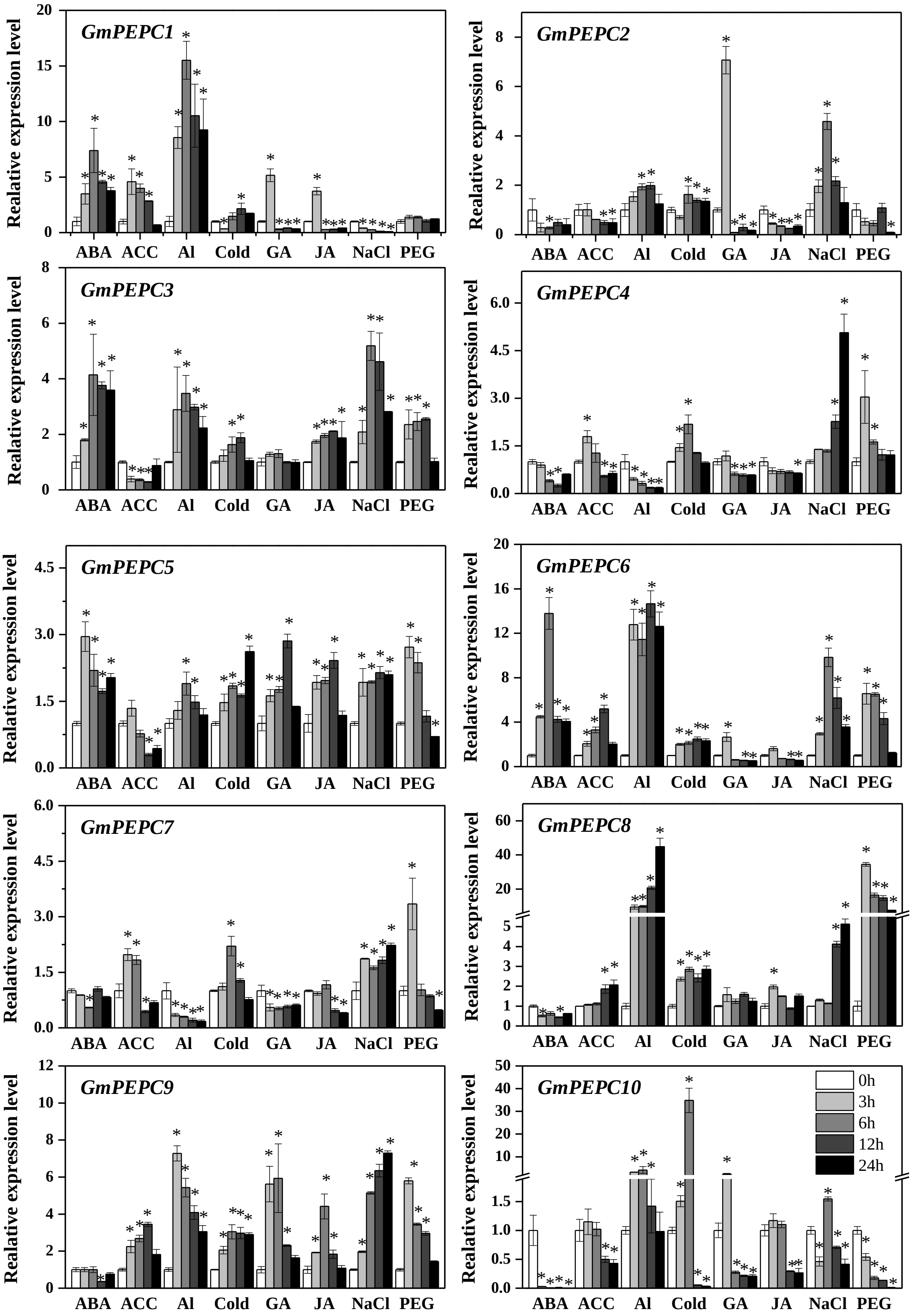
GmPEPC1
GmPEPC2
GmPEPC3
GmPEPC4
GmPEPC5
GmPEPC6
GmPEPC7
GmPEPC8
GmPEPC9
GmPEPC10
Realative expression level
ABA ACC Al Cold GA JA NaCl PEG
0h
3h
6h
12h
24h

Figure S5. Patterns of four *PPCK* genes expression in soybean leaves (A) and roots (B) under different stresses and hormone treatments. The 14-d soybean seedlings were treated with 100 μM ABA (abscisic acid), 100 μM ACC (1-aminocyclopropane-1-carboxylic acid), 25 μM $AlCl_3$ (pH 4.3), 4 ℃ cold, 100 μM GA (gibberellins), 100 μM JA (jasmonic acid), 200 mM NaCl, and 20 % (w/v) PEG (polyethylene glycol) 6000. The relative expression levels were analyzed by qRT-PCR using the house-keeping gene *GmRP15* as the internal control. Values are means ± SD of three biological replicates. Statistical significance of differences between control and treated groups was analyzed using Student's t-test (* indicates $P < 0.05$).

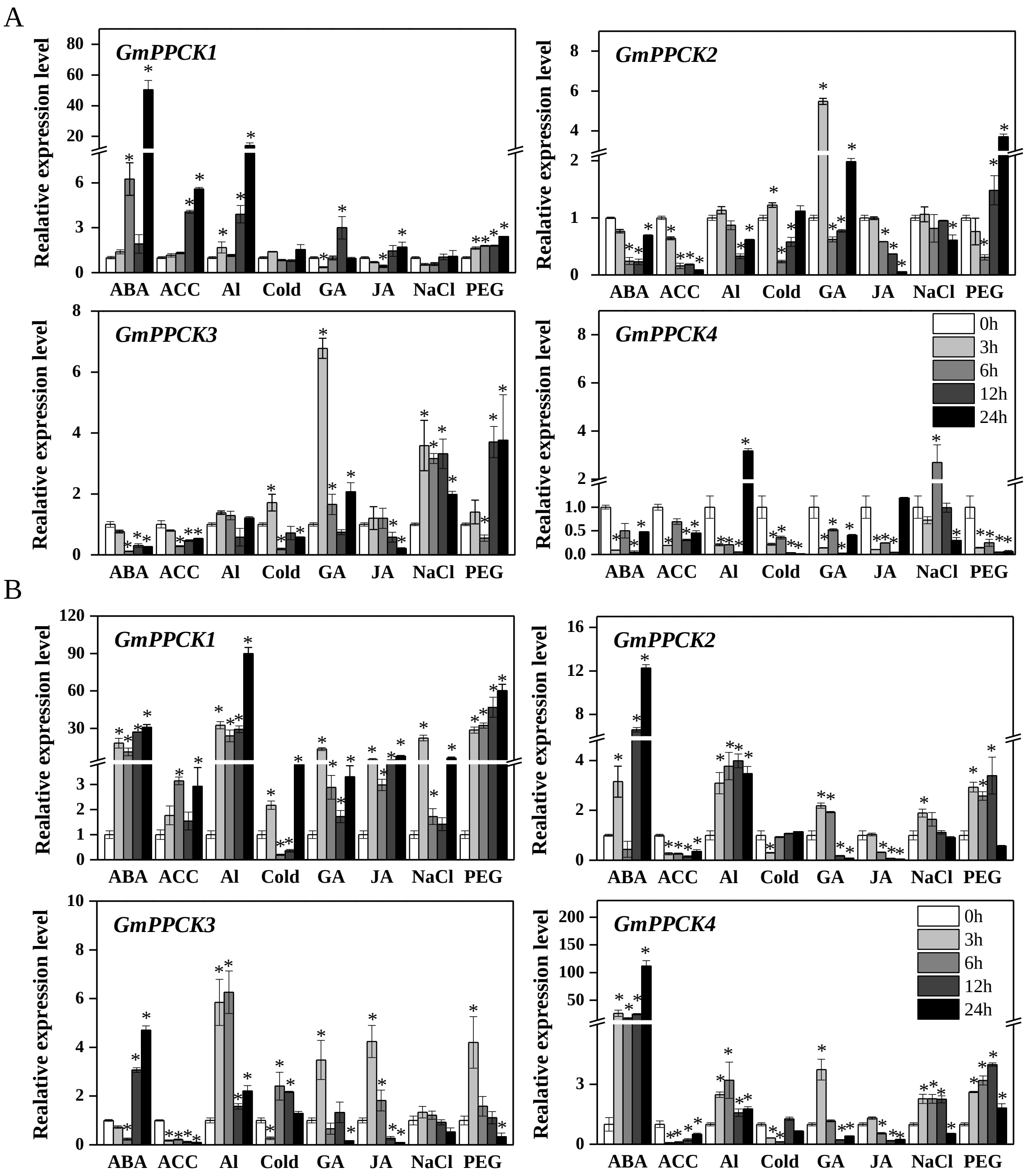
A
B
GmPPCK1
GmPPCK2
GmPPCK3
GmPPCK4
Realative expression level
ABA ACC Al Cold GA JA NaCl PEG
0h
3h
6h
12h
24h
